# Brd4 modulates diet-induced obesity via PPAR**γ**-dependent Gdf3 expression in adipose tissue macrophages

**DOI:** 10.1172/jci.insight.143379

**Published:** 2021-04-08

**Authors:** Xiangming Hu, Xingchen Dong, Guo Li, Yanheng Chen, Jinjing Chen, Xiaoxin He, Hao Sun, Dong-Hyun Kim, Jongsook Kim Kemper, Lin-Feng Chen

**Affiliations:** 1 Fujian Key Laboratory for Translational Research in Cancer and Neurodegenerative Diseases, Institute for Translational Medicine, School of Basic Medical Sciences, Fujian Medical University, Fuzhou, China.; 2 Department of Biochemistry,; 3 Department of Molecular and Integrative Physiology, and; 4 Carl R. Woese Institute for Genomic Biology, University of Illinois at Urbana-Champaign, Urbana, Illinois, USA.

**Keywords:** Metabolism, Macrophages, Obesity, Translation

## Abstract

Macrophage-mediated inflammatory response has been implicated in the pathogenesis of obesity and insulin resistance. Brd4 has emerged as a key regulator in the innate immune response. However, the role of Brd4 in obesity-associated inflammation and insulin resistance remains uncharacterized. Here, we demonstrated that myeloid lineage-specific Brd4 knockout (*Brd4*-CKO) mice were protected from high-fat diet–induced (HFD-induced) obesity with less fat accumulation, higher energy expenditure, and increased lipolysis in adipose tissue. *Brd4*-CKO mice fed a HFD also displayed reduced local and systemic inflammation with improved insulin sensitivity. RNA-Seq of adipose tissue macrophages (ATMs) from HFD-fed WT and *Brd4*-CKO mice revealed that expression of antilipolytic factor Gdf3 was significantly decreased in ATMs of *Brd4*-CKO mice. We also found that Brd4 bound to the promoter and enhancers of *Gdf3* to facilitate PPARγ-dependent Gdf3 expression in macrophages. Furthermore, Brd4-mediated expression of Gdf3 acted as a paracrine signal targeting adipocytes to suppress the expression of lipases and the associated lipolysis in cultured cells and mice. Controlling the expression of Gdf3 in ATMs could be one of the mechanisms by which Brd4 modulates lipid metabolism and diet-induced obesity. This study suggests that Brd4 could be a potential therapeutic target for obesity and insulin resistance.

## Graphical abstract

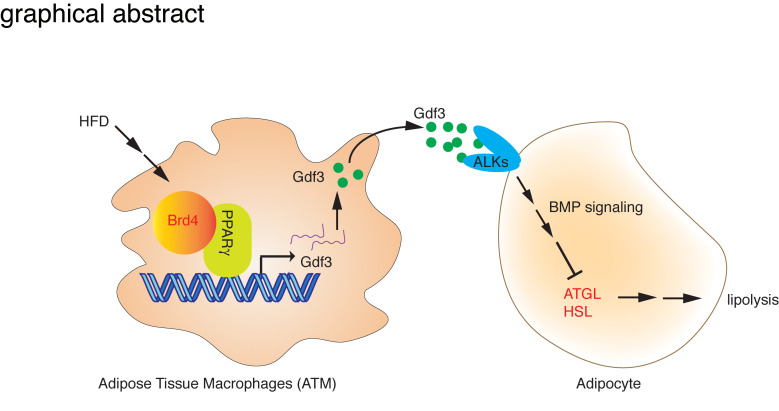

## Introduction

Obesity is characterized by increased fat accumulation associated with a chronic low-grade inflammation that plays a key role in promoting obesity-associated insulin resistance (1). Cells of the innate immune system, including adipose tissue macrophages (ATMs), produce proinflammatory cytokines, and various factors that trigger the chronic inflammation, promote fat accumulation, and impair insulin signaling, contributing to the development of obesity and the onset of type 2 diabetes mellitus (2, 3). A paracrine loop between ATMs and adipocytes is crucial for maintaining adipose tissue homeostasis in health as well as the pathogenesis of obesity-associated metabolic diseases (2, 3).

Fat accumulation results from excessive triglycerides (TGs) storage in white adipose tissue (WAT) that occurs when TG synthesis exceeds TG hydrolysis, the lipolysis (4). The major enzymes for TG hydrolysis in adipose tissue consist of adipose TG lipase (ATGL) and hormone-sensitive lipase (HSL). ATGL hydrolyzes TG into diglyceride, which is subsequently hydrolyzed by HSL and monoglyceride lipase (MGL) into glycerol and free fatty acids (FFAs; refs. 5, 6). ATGL is the first and rate-limiting enzyme in TG hydrolysis and plays a critical role in lipid and energy metabolism, and its dysfunction is associated with metabolic diseases (7). Therefore, the expression and activity of ATGL are tightly regulated in response to energy demands or nutritional status by several hormones, including catecholamine and insulin (4, 8). For example, the mRNA and protein levels of ATGL were increased in adipose tissues upon fasting to stimulate lipolysis (9). Catecholamine activates ATGL for lipolysis via PKA-mediated phosphorylation of PLIN1 (10). On the other hand, insulin has been shown to directly target adipocytes to suppress ATGL expression and lipolysis (5, 11). Recent studies also indicate that the expression of ATGL can be regulated by growth differentiation factor 3 (Gdf3), an antilipolytic factor produced from CD11c^+^ ATMs (12, 13).

Although both ATMs and adipocytes express Gdf3, ATMs are considered the main source of Gdf3, and its expression in ATMs can be regulated by insulin or NLRP3 inflammasome (12, 13). Low levels of insulin stimulate the expression of Gdf3, which suppresses ATGL expression and lipolysis in adipose tissue (13). In addition, inflammasome-mediated activation of Gdf3 in ATMs suppresses catecholamine-induced ATGL activation and lipolysis in adipocytes of aging mice (12). Gdf3 acts as a ligand of ALK7 (activin receptor-like kinase 7) to decrease the activity of PPARγ or C/EBPα in differentiated adipocytes, leading to the suppression of ATGL-mediated lipolysis with increased adipocyte size and TG content (12–15). PPARγ has been shown to bind to the enhancers of *Gdf3* and activate its transcription in macrophages to control tissue repair during skeletal muscle regeneration (16). However, how the expression of Gdf3 is regulated in ATMs remains largely undetermined.

Brd4 belongs to the bromodomain extra terminal (BET) family and has emerged as an important epigenetic regulator in inflammatory gene expression and cancer development (17, 18). By binding to acetylated histones or nonhistone proteins, Brd4 regulates gene transcription by recruiting different transcription components, such as Mediator and P-TEFb, to activate RNA polymerase II–dependent (RNAPII-dependent) transcription (17, 18). Emerging evidence suggests that Brd4 also regulates metabolism in specific tissues (19, 20). For example, inhibition of Brd4 has been shown to reduce systemic inflammation and abrogate inflammation-related metabolic diseases, including atherosclerosis and liver fibrosis (21–23). Brd4 also regulates insulin content in pancreatic cells and adipogenesis in adipocytes via P-TEFb-mediated gene expression (24–27). Our recent study demonstrates that Brd4 plays a critical role in innate immune response by modulating the proinflammatory gene expression in macrophages (28). Although ATMs have been shown to be the major source of inflammatory mediators that are linked to obesity and insulin resistance (1), the possible pathological functions of macrophage Brd4 in metabolic diseases remain largely elusive.

In an effort to explore the potential role of Brd4 in ATMs and in the development of obesity and insulin resistance, we found that mice with deficiency of Brd4 in myeloid lineage-specific cells were protected from high-fat diet–induced (HFD-induced) obesity, inflammation, and insulin resistance with increased energy expenditure and enhanced lipolysis in adipose tissue. Deficiency of Brd4 was associated with reduced expression of Gdf3 in ATMs. Brd4 occupied the promoter and enhancers of Gdf3 to facilitate PPARγ-dependent expression of Gdf3, which in turn suppressed ATGL expression and lipolysis in adipocytes, resulting in the increased fat accumulation and insulin resistance. Our study reveals, what we believe to be, a function of Brd4 in lipid metabolism and obesity, indicating that Brd4 could be a potential therapeutic target to prevent and treat obesity and the associated metabolic diseases.

## Results

**Figure 1:**
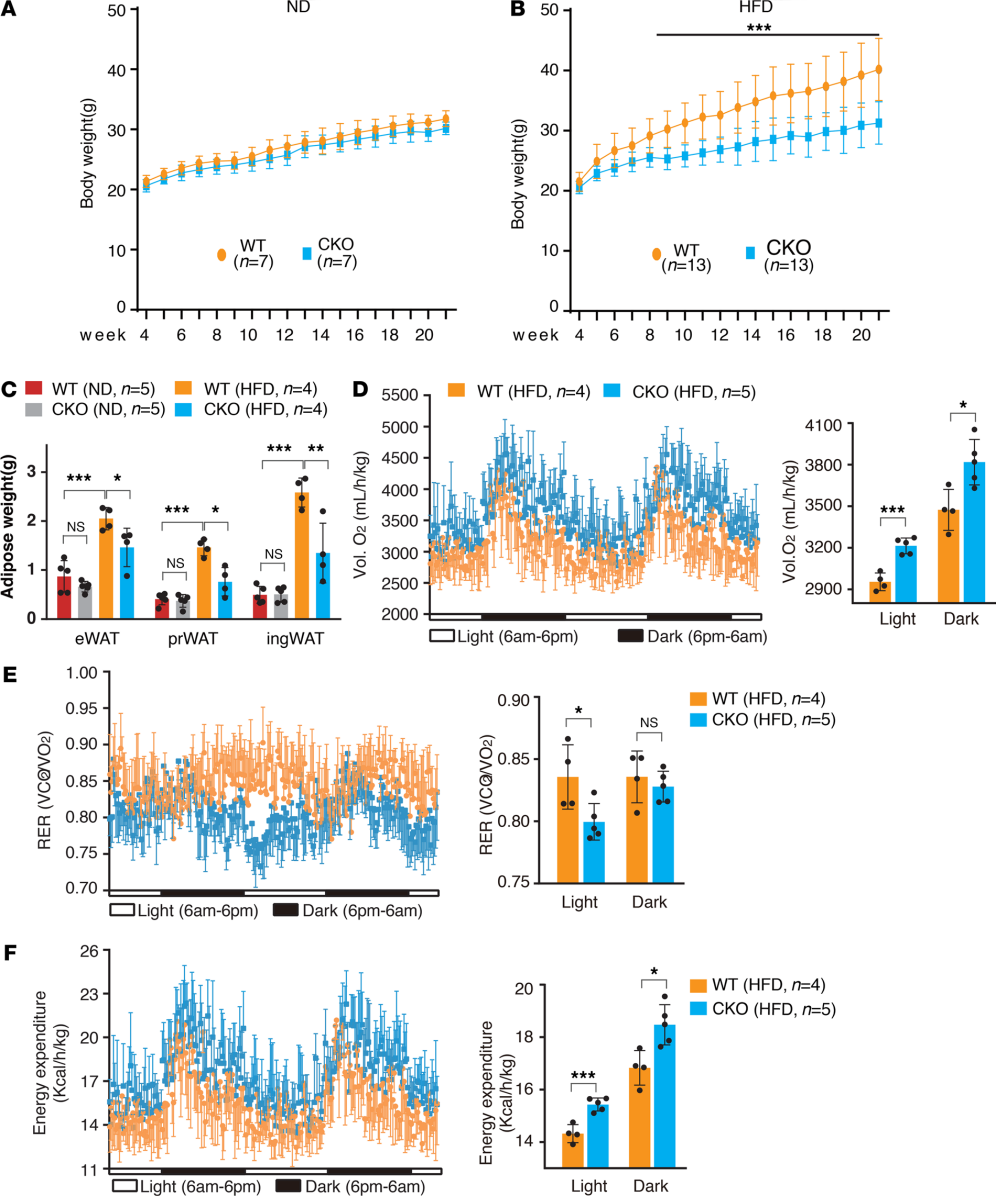
Mice with myeloid lineage-specific deletion of Brd4 were protected from diet-induced obesity with increased energy expenditure. Weight gain of WT and *Brd4*-CKO mice fed a ND (**A**) or a HFD (**B**) from 4 to 21 weeks of age. (**C**) The weights of adipose tissues of WT or *Brd4*-CKO mice after 17 weeks on a ND or a HFD. Metabolic studies of WT or *Brd4*-CKO mice fed a HFD measured over 48 hours by CLAMS. O_2_ consumption (**D**), RER (**E**), and energy expenditure measurements (**F**) were performed on mice after 3 weeks on a HFD. RER was calculated as the ratio of the volume of CO_2_ production to the volume of O_2_ consumption. Data are mean and SD and are determined by an unpaired 2-tailed Student’s *t* test (**A** and **B**) or 1-way ANOVA (**C**–**F**). *n =* 4–13 mice as indicated. *P ≤ 0.05, **P ≤ 0.01, ***P ≤ 0.001, ns, statistically not significant. *Brd4*-CKO, myeloid lineage-specific Brd4 knockout; HFD, high-fat diet–induced; ND, normal diet; RER, respiratory exchange ratio.

*Myeloid lineage–specific Brd4 knockout* (*Brd4-CKO) mice were protected from diet-induced obesity with decreased respiratory exchange ratio but increased energy expenditure*. To explore the potential role of Brd4-mediated innate immune response in obesity, we used a mouse model of obesity with HFD using WT (*Brd4^fl/fl^*) and *Brd4*-CKO (*Brd4^fl/fl^*-*LysM^Cre^*) mice. Although WT and *Brd4*-CKO mice fed a normal diet (ND) for 17 weeks had similar body weight (Figure 1A), *Brd4*-CKO mice fed a HFD had significantly less body weight than WT mice on the same HFD (Figure 1B). Compared with obese WT mice, *Brd4*-CKO mice exhibited reduced WAT, including epididymal WAT (eWAT), perirenal WAT (prWAT), and inguinal WAT after 20 weeks of HFD feeding (Figure 1C and Supplemental Figure 1; supplemental material available online with this article; https://doi.org/10.1172/jci.insight.143379DS1). There was no significant weight difference in heart, lung, kidney, and spleen between HFD-fed WT and HFD-fed *Brd4*-CKO mice (Supplemental Figure 2, left-hand side of panel), except a reduced liver weight in *Brd4*-CKO mice, whereas ND-fed WT and *Brd4*-CKO mice had similar liver weight (Supplemental Figure 2, right-hand side of panel).

To understand the reduced body weight in HFD-fed *Brd4*-CKO mice, we analyzed whole-body energy homeostasis in WT and *Brd4*-CKO mice fed the same HFD using indirect calorimetry. We measured several metabolic parameters in vivo in WT and *Brd4*-CKO mice after 3 weeks of HFD feeding using the Comprehensive Laboratory Animal Monitoring System (CLAMS). During the 48-hour light and dark phases, there was no significant difference in daily locomotor activity and food intake between WT and *Brd4*-CKO mice (Supplemental Figure 3, A and B). However, the oxygen consumption (VO_2_) and CO_2_ release (VCO_2_) were increased in *Brd4*-CKO mice compared with WT mice (Figure 1D and Supplemental Figure 3C) with a reduced respiratory exchange ratio (RER) in *Brd4*-CKO mice during the light phase (Figure 1E). Compared with WT rest mice, the RER from rest *Brd4*-CKO mice was reduced from approximately 0.85 to 0.8, indicating that more fatty acids were used as fuel in *Brd4*-CKO mice (Figure 1E). Furthermore, the overall energy expenditure was significantly higher, with at least a 7.7% increase during the light (7.7%) and dark (9.7%) phases in the *Brd4*-CKO mice fed a HFD (Figure 1F). These data suggest that energy expenditure was increased in HFD-fed *Brd4*-CKO mice, likely with increased lipids serving as the energy substrates.

### Brd4-CKO mice displayed improved insulin sensitivity in obesity.

**Figure 2:**
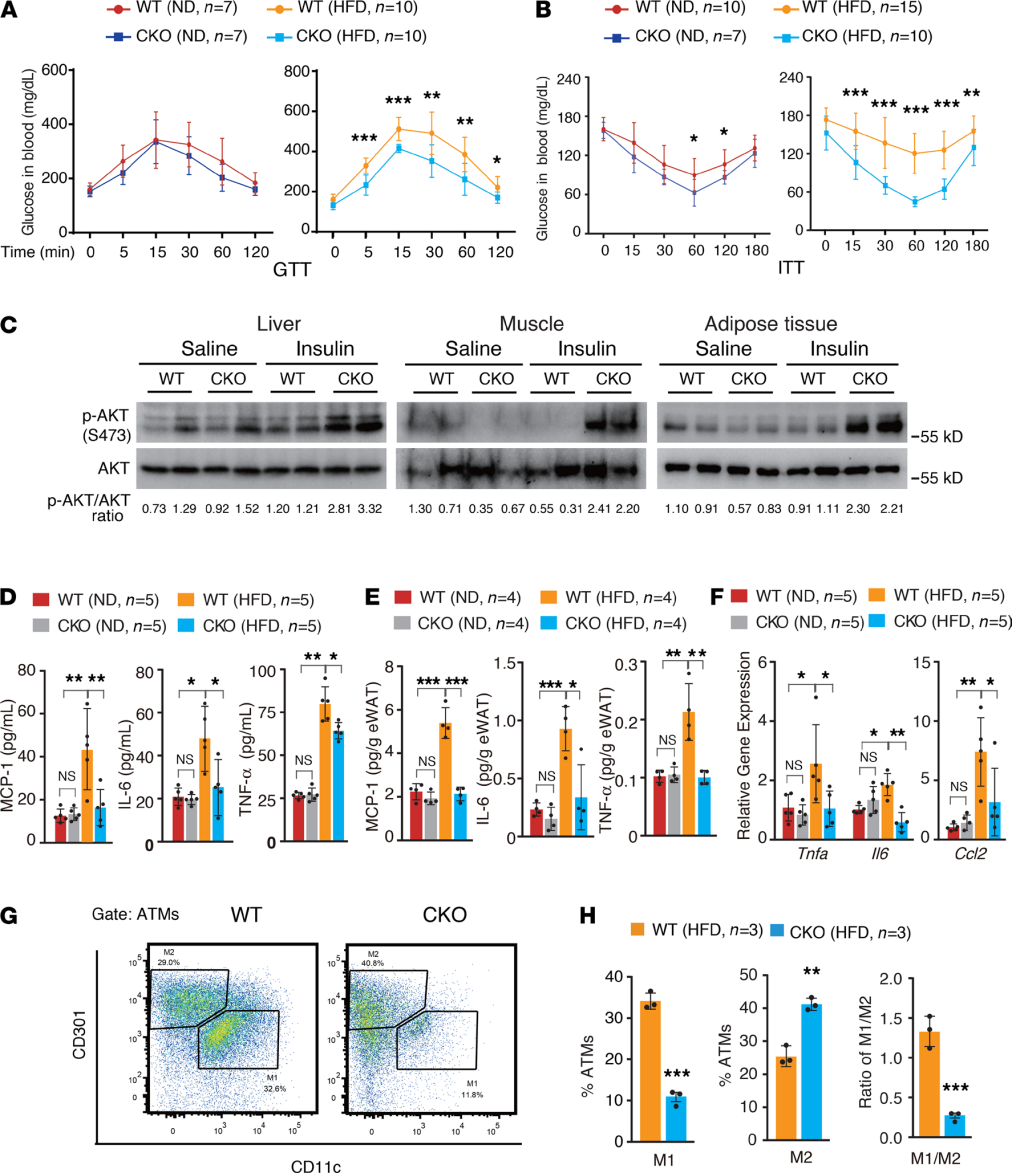
*Brd4*-CKO mice had improved insulin sensitivity and reduced inflammation in response to HFD. Plasma glucose levels during GTT (**A**) and ITT (**B**) in WT or *Brd4*-CKO mice fed a ND or a HFD for 18 weeks. (**C**) Immunoblotting of p-AKT and total AKT in liver, muscle, and adipose tissue of overnight fasting WT or *Brd4*-CKO mice fed a HFD for 20 weeks with saline or insulin (1 unit/kg) injection. Ratios of p-AKT to total AKT were determined by densitometry using ImageJ. (**D**) ELISA assay of MCP-1, IL-6, and TNF-α levels in plasma of WT or *Brd4*-CKO mice fed a ND or a HFD for 20 weeks. (**E**) ELISA assay of MCP-1, IL-6, and TNF-α levels in eWAT of WT or *Brd4*-CKO mice fed a ND or a HFD for 20 weeks. (**F**) mRNA levels of *Tnfa*, *Il6*, and *Mcp1* were measured by real-time PCR in eWAT of WT or *Brd4*-CKO mice fed a ND or a HFD for 20 weeks. (**G**) M1 (CD11c^+^ CD301^-^) and M2 (CD11c^-^ CD301^+^) macrophages in ATMs of WT and *Brd4*-CKO mice fed a HFD. Gating strategies to determine M1-type and M2-type ATMs are depicted in Methods. (**H**) Percentages of M1-type, M2-type ATMs and the ratio of M1 and M2 ATMs in eWAT of WT and *Brd4*-CKO mice fed a HFD. Data are mean and SD and are determined by an unpaired 2-tailed Student’s *t* test (**A**, **B**, and **H**) or 1-way ANOVA (**D**–**F**). *n =* 3–15 mice as indicated. *P < 0.05, **P < 0.01, ***P < 0.001, ns, statistically not significant. *Brd4*-CKO, myeloid lineage-specific Brd4 knockout; HFD, high-fat diet–induced; ND, normal diet; GTT, glucose tolerance test; ITT, insulin tolerance test; eWAT, epididymal WAT; ATMs, adipose tissue macrophages.

Because HFD-induced obese mice develop glucose intolerance and insulin resistance (1), we next compared the insulin sensitivity of WT and *Brd4*-CKO mice fed a ND or a HFD by the glucose tolerance test (GTT) and insulin tolerance test (ITT). Both WT and *Brd4*-CKO mice, fed either a ND or a HFD, had similar fasting blood glucose levels (Supplemental Figure 4A). After i.p. injection of a glucose bolus, WT and *Brd4*-CKO mice fed a ND had a similar response (Figure 2A, left-hand side of panel). However, HFD-fed *Brd4*-CKO mice were less hyperglycemic compared with obese WT mice at various time points (Figure 2A, right-hand side of panel). When we measured the insulin sensitivity in these mice with ITT, we observed that *Brd4*-CKO mice fed a ND were slightly more sensitive to insulin than WT mice (Figure 2B, left-hand side of panel). On a HFD, *Brd4*-CKO mice were much more sensitive to insulin than obese WT mice (Figure 2B, right-hand side of panel) even though the plasma levels of insulin were higher in obese WT mice than those in lean *Brd4*-CKO mice (Supplemental Figure 4B). These data suggest that deletion of *Brd4* in myeloid cells improved insulin sensitivity in mice fed a HFD.

Insulin resistance occurs in metabolic tissues, including the liver, muscle, and adipose tissue (1, 29). To further determine the improved insulin sensitivity in *Brd4*-CKO mice, we measured the phosphorylation of AKT, which represents the activation of AKT and insulin signaling, in these various tissues (30). After overnight fasting, we injected HFD-fed obese WT mice and *Brd4*-CKO mice with saline or insulin through the inferior vena cava and quickly removed liver, muscle, and adipose tissue for biochemical analyses. Consistent with the insulin insensitivity in obese WT mice, the levels of phosphorylated AKT were not changed or slightly reduced in response to insulin injection (Figure 2C). Conversely, the levels of phosphorylated AKT were dramatically increased in liver, muscle, and adipose tissue of *Brd4*-CKO mice after insulin injection compared with WT mice (Figure 2C). These findings confirm that *Brd4*-CKO mice are more sensitive to insulin than WT mice fed a HFD.

### Brd4-CKO mice exhibited reduced inflammation after HFD feeding.

Obesity is often associated with low-grade inflammation, which contributes to the development of insulin resistance (31, 32). We thus examined the plasma levels of proinflammatory cytokines or chemokines in WT and *Brd4*-CKO mice fed a ND or a HFD. The levels of plasma MCP-1, IL-6, and TNF-α were not changed in WT and *Brd4*-CKO mice fed a ND (Figure 2D). In contrast, plasma levels of MCP-1, IL-6, and TNF-α were elevated in HFD-fed obese WT mice (Figure 2D). However, the levels of these cytokines or chemokines were decreased in *Brd4*-CKO mice fed the same HFD (Figure 2D). These results indicate that the myeloid-specific deletion of *Brd4* reduced HFD-triggered inflammation. Because WAT is the key site mediating systemic inflammation during obesity (29, 33), we evaluated the inflammation in WAT of WT and *Brd4*-CKO mice. Consistent with the reduced systemic inflammation in HFD-fed *Brd4*-CKO mice, the levels of MCP-1, IL-6, and TNF-α were found to be decreased in adipose tissue of *Brd4*-CKO mice compared with those of WT mice fed a HFD (Figure 2E). Furthermore, the mRNA levels of *Tnf*, *Il6,* and *Mcp1* were decreased in the adipose tissue of HFD-fed *Brd4*-CKO mice (Figure 2F). Altogether, these data demonstrate that deletion of *Brd4* in myeloid cells reduced diet-induced chronic inflammatory response.

A transition in macrophage polarization from an alternative M2 activation state to a classical M1 activation state has also been indicated as a molecular mechanism for the HFD-induced adipose tissue inflammation and insulin resistance (34). Therefore, we examined the effect of Brd4-deficiency on the polarization of ATMs. FACS analysis (35) of M1 (CD45^+^, CD11b^+^, F4/80^+^, CD11c^+^, CD301^-^) and M2 (CD45^+^, CD11b^+^, F4/80^+^, CD11c^-^, CD301^+^) macrophages from SVCs of eWAT in HFD-fed WT and *Brd4*-CKO mice demonstrated that there was an increased percentage of M2 but reduced percentage of M1 macrophages in *Brd4*-CKO mice (Figure 2, G and H), although the total amount of ATMs was decreased in *Brd4*-CKO mice (Supplemental Figure 5). The ratio of M1 to M2 macrophages was dramatically reduced in *Brd4*-CKO mice (Figure 2H), suggesting that Brd4 might also regulate the polarization of ATMs, favoring a proinflammatory status of macrophages.

### ATMs from Brd4-CKO mice had decreased expression of Gdf3.

**Figure 3:**
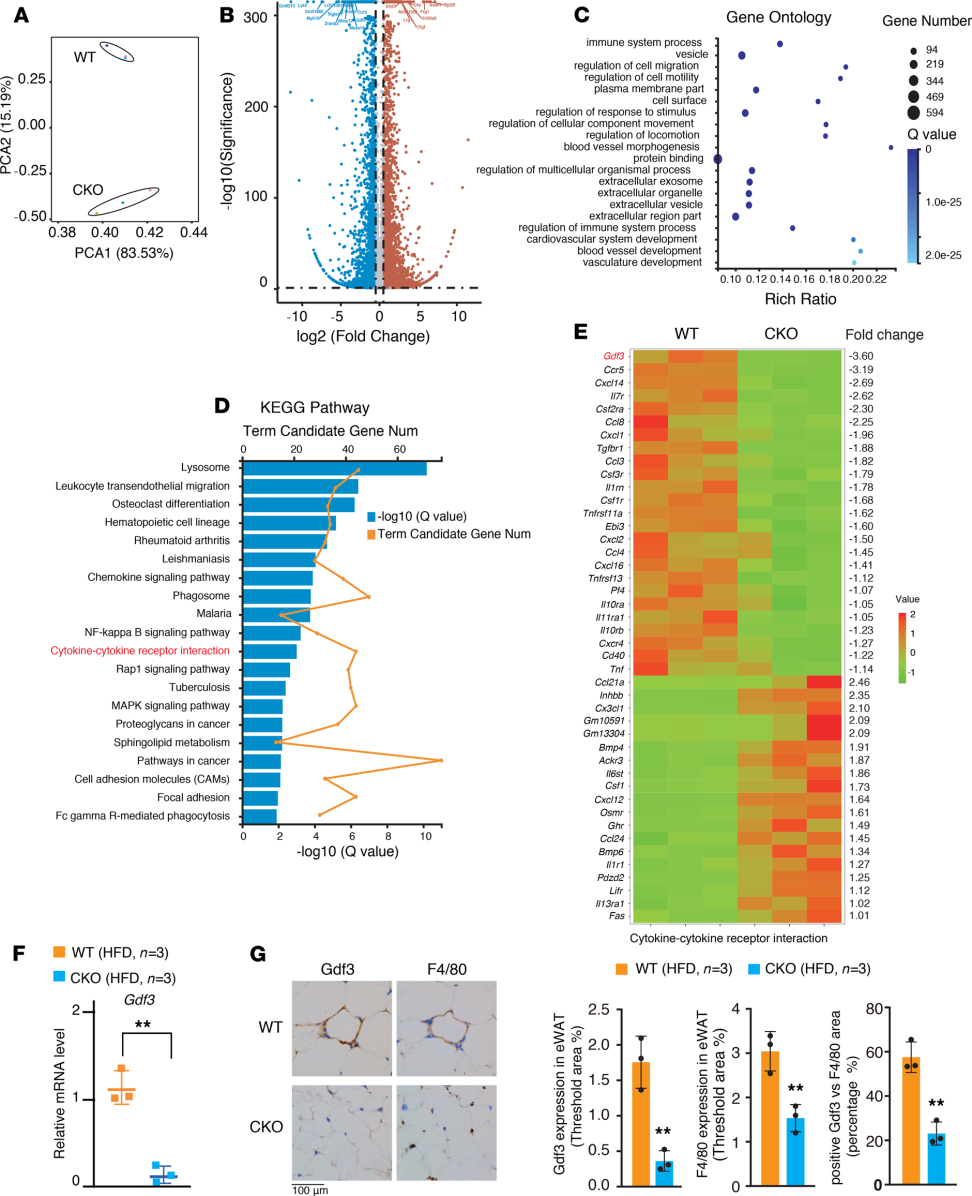
*Brd4*-CKO mice had reduced expression of Gdf3 in ATMs. (**A**) PCA of RNA-Seq data derived from CD11b^+^ ATMs of WT and *Brd4*-CKO mice fed a HFD for 20 weeks. (**B**) Volcano plot of mRNA-Seq analysis in ATMs as indicated in **A**. Brown dots represent genes increased in ATMs of *Brd4*-CKO mice vs. WT mice (fold change ≥ 2, adjusted *P* value ≤ 0.001, calculated by raw count value). Blue dots represent genes decreased in ATMs of *Brd4*-CKO mice vs. WT mice (fold change ≥ 2, adjusted *P* value ≤ 0.001, calculated by raw count value). Gray dots represent genes without significantly altered expression. Clusters of significantly altered genes (fold change ≥ 2, adjusted *P* value ≤ 0.001, calculated by raw count value) were identified using gene ontology terms (**C**) and KEGG pathways (**D**). (**E**) Heat map of the relative expression levels (scaled *Z*-score) of cytokine-cytokine receptor interaction-related genes clustered in (**D**). (**F**) mRNA levels of Gdf3 in CD11b^+^ ATMs isolated from WT or *Brd4*-CKO mice fed a HFD for 20 weeks. (**G**) Left panel: Gdf3 or F4/80 IHC staining of eWAT of WT or Brd4-CKO mice fed a HFD for 20 weeks. Right panel: statistical analysis of Gdf3-positive or F4/80-positive area percentage, the ratio of Gdf3-positive cells in F4/80-positive cells in eWAT of WT and *Brd4*-CKO mice fed a HFD. Data are mean and SD and are determined by an unpaired 2-tailed Student’s *t* test. *n =* 3 mice. **P < 0.01. *Brd4*-CKO, myeloid lineage-specific Brd4 knockout; HFD, high-fat diet–induced; eWAT, epididymal WAT; ATMs, adipose tissue macrophages; PCA, principal component analysis.

To explore the mechanism by which myeloid-specific ablation of *Brd4* reduced adipose tissue mass and inflammation, we performed an RNA-Seq of CD11b^+^ macrophages isolated from adipose tissue of HFD-fed WT and HFD-fed *Brd4*-CKO mice. Principal component analysis based on variable genes demonstrated segregation of the samples by polarization state (Figure 3A). Using fold-change difference greater than or equal to 2 and adjusted *P* value less than or equal to 0.001 as cutoffs to determine differentially expressed genes (DEGs), we observed that *Brd4* deficiency resulted in the downregulation of 673 genes and the upregulation of 588 genes in ATMs (Figure 3B and Supplemental Figure 6).

Consistent with the previous finding that Brd4 acts as a key regulator of innate immune response (28), gene ontology and KEGG pathway analysis revealed significant enrichments for genes associated with immune system process, including cytokine-cytokine receptor interaction, chemokine signaling, and NF-κB signaling pathways (Figure 3, C and D). We further examined the genes in the cytokine-cytokine receptor interaction because we suspected that ATMs regulate the functionality of adipocytes in a paracrine manner. Of interest, *Gdf3*, which encodes a cytokine inhibiting lipolysis (12, 13), was the most decreased genes in ATMs of HFD-fed *Brd4*-CKO mice (Figure 3E). The decreased expression of *Gdf3* was further confirmed by quantitative PCR (qPCR) in ATMs isolated from HFD-fed WT and HFD-fed *Brd4*-CKO mice (Figure 3F). IHC staining of eWAT for Gdf3 also revealed that Gdf3 was highly expressed around the enlarged adipocytes of WT obese mice (Figure 3G). In contrast, Gdf3 levels were significantly decreased in eWAT of the lean *Brd4*-CKO mice (Figure 3G). Importantly, Gdf3-positive cells predominantly overlaid with F4/80 staining positive ATMs, which displayed the crown-like structures (Figure 3G), supporting the notion that ATMs rather than adipocytes are the prominent source of Gdf3 in adipose tissue (13). The percentage of Gdf3-expressing cells from F4/80-positive cells was significantly reduced from approximately 60% in adipose tissue of WT mice to approximately 20% in *Brd4*-CKO mice (Figure 3G). These data demonstrate that Brd4 is essential for the expression of Gdf3 in ATMs.

**Figure 4:**
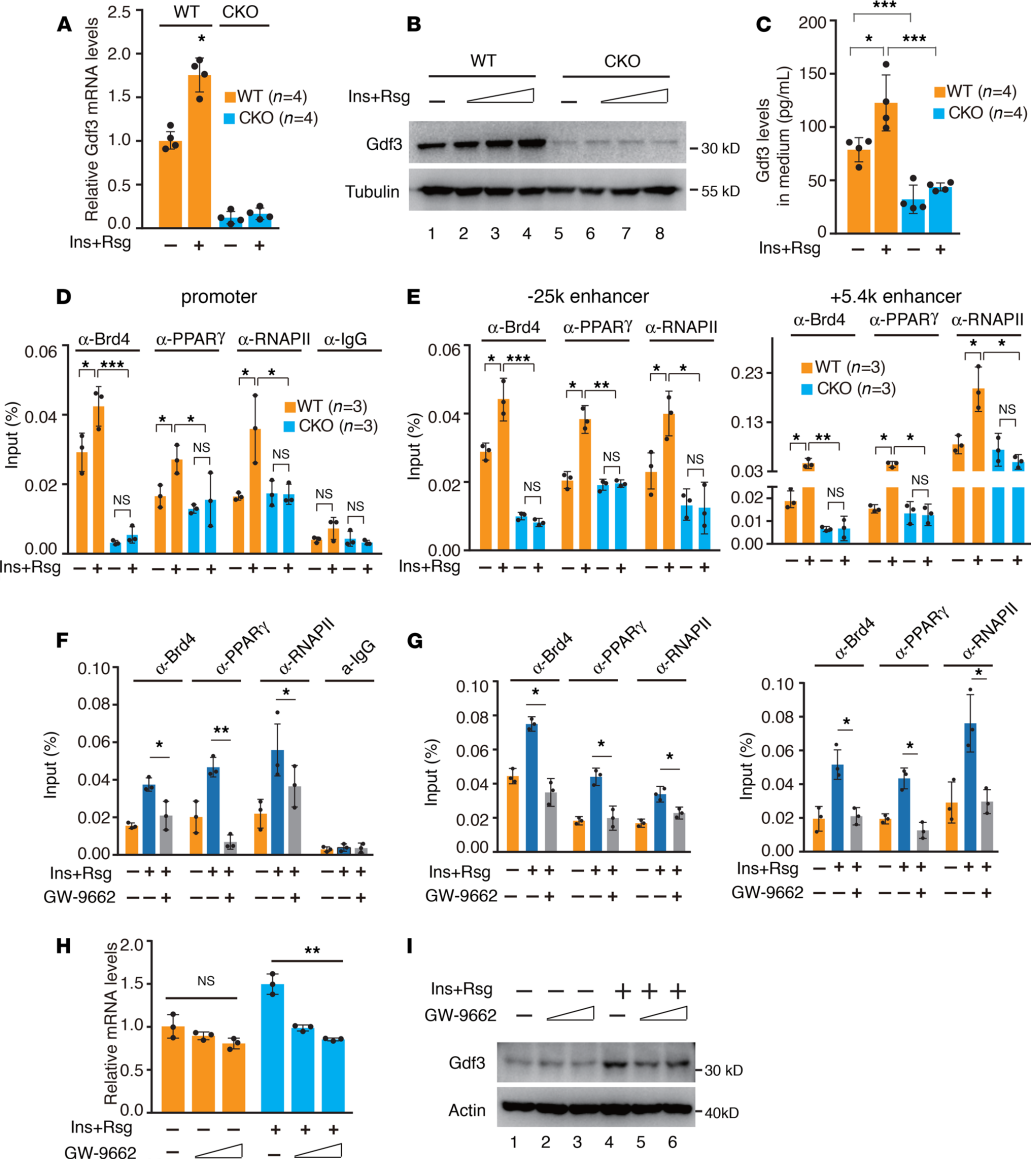
Brd4 cooperated with PPARγ to regulate the expression of Gdf3 in macrophages. (**A**) WT BMDMs or *Brd4*-deficient BMDMs were treated with or without 1 nM Ins and 50 μM Rsg for 12 hours; Gdf3 mRNA levels were analyzed by real-time PCR. (**B**) WT BMDMs or *Brd4*-deficient BMDMs were treated with or without 0.1, 1, 10 nM Ins, and 50 μM Rsg for 16 hours; Gdf3 protein levels were analyzed by immunoblotting. (**C**) WT BMDMs or *Brd4*-deficient BMDMs were treated with or without Ins and Rsg as in **A** for 16 hours; Gdf3 protein levels in the medium were analyzed by ELISA. WT BMDMs or *Brd4*-deficient BMDMs were treated with or without Ins and Rsg as in **A** for 12 hours. ChIP assays were performed using antibodies against Brd4, PPARγ, RNAPII, and IgG and probed for the promoter (**D**) and enhancers (–25K and +5.4K) (**E**) of Gdf3. (**F** and **G**) WT BMDMs were pretreated with or without PPARγ antagonist GW6992 (10 μM) for 1 hour, followed by the stimulation with Ins plus Rsg for 12 hours. ChIP assays were performed using antibodies against Brd4, PPARγ, RNAPII, and IgG and probed for the promoter (**F**) and enhancers (-25K and +5.4K) (**G**) of Gdf3. WT BMDMs were pretreated with GW6992 (10 μM or 30 μM) for 1 hour, followed by the stimulation with Ins plus Rsg for 12 hours (**H**) or 24 hours (**I**). mRNA (**H**) and protein (**I**) levels of Gdf3 were analyzed by RT-PCR or immunoblotting, respectively. Data are mean and SD and are determined by 1-way ANOVA. *n =* 3-4 culture dishes from 3–4 independent experiments. *P ≤ 0.05, **P ≤ 0.01, ***P ≤ 0.001, ns, statistically not significant. BMDMs, BM-derived macrophages; Ins, insulin; Rsg, rosiglitazone.

*Brd4 regulated the expression of Gdf3 via PPAR*γ*-dependent occupancy at the promoter and enhancers*. To further determine how Brd4 regulates the expression of *Gdf3*, we elected to use BM-derived macrophages (BMDMs), a well-established in vitro model system to interrogate the regulatory mechanism exerted by Brd4 on *Gdf3*. The expression of *Gdf3* in ATMs is activated by a low concentration of insulin (13). In addition, transcription of *Gdf3* is regulated by PPARγ in macrophages (16). Therefore, we treated BMDMs with insulin and PPARγ agonist Rosiglitazone (Rsg) to induce the expression of *Gdf3*. As expected, 1 nM of insulin and 50 μM of Rsg increased the transcription of *Gdf3* in WT BMDMs, whereas the expression was not changed under separate treatment. (Figure 4A and Supplemental Figure 7). However, the mRNA levels of *Gdf3* were significantly reduced in *Brd4*-deficient BMDMs with or without insulin and Rsg stimulation (Figure 4A), indicating that the basal and the inducible expression of Gdf3 was regulated by Brd4. In addition, the cellular protein levels of Gdf3 were increased by insulin and Rsg treatment but were dramatically reduced in *Brd4*-deficient BMDMs with or without stimulation (Figure 4B). Importantly, the secreted forms of Gdf3 were similarly decreased in the culture media from *Brd4*-deficient BMDMs compared with those of WT BMDMs (Figure 4C). Altogether, these data indicate that Brd4 regulates both the basal and the induced expression of *Gdf3* in macrophages.

Brd4 binds to the promoters and enhancers to regulate gene expression (20). Because the expression of *Gdf3* in macrophages is regulated by PPARγ (16), we suspected that Brd4 cooperated with PPARγ to facilitate the expression of *Gdf3*. We first performed ChIP assay to examine the recruitment of Brd4, PPARγ, and RNAPII to the promoter of *Gdf3* in WT BMDMs and *Brd4*-deficient BMDMs. In WT BMDMs, insulin and Rsg stimulated the binding of Brd4 and PPARγ to the promoter of *Gdf3* (Figure 4D), which contains a PPRAγ binding site (Supplemental Figure 8). Insulin and Rsg also stimulated the binding of RNAPII to the promoter in WT BMDMs (Figure 4D). Interestingly, insulin and Rsg didn’t enhance the binding of PPARγ on the promoter of *Gdf3* in *Brd4*-deficient BMDMs as in WT BMDMs, whereas deficiency of Brd4 had little effect on the basal PPARγ recruitment (Figure 4D). These data indicate that Brd4 might have been involved in the stimulus-dependent recruitment of PPARγ to the promoter. Consistent with Brd4’s role in signal-dependent RNAPII recruitment (36), insulin and Rsg failed to stimulate the recruitment of RNAPII to the promoter in *Brd4*-deficient BMDMs (Figure 4D).

PPARγ activates the transcription of *Gdf3* by binding to its enhancers and Brd4 can also regulate gene expression via enhancers (16, 20), raising a possibility that Brd4 could also cooperate with PPARγ to stimulate Gdf3 expression via enhancers. When we analyzed the available ChIP-Seq database (GSE109131 for Brd4, GSE21314 for PPARγ, and GSE106701 for H2K27Ac all from NCBI) from BMDMs for Brd4 enrichment on the locus of *Gdf3*, we found that Brd4 was enriched in several potential enhancer regions of Gdf3, including +38K, +7.3K, +5.4K, –25K, –28K, –44K, and –47K, which are associated with enrichment of H3K27Ac, a histone mark for active enhancer (Supplemental Figure 9). Among these 7 enhancers, 4 (+7.3K, –25K, –44K, and –47K) have been reported to be occupied and likely regulated by PPARγ (16). We next examined the recruitment of Brd4, PPARγ, and RNAPII to 2 different representative enhancers (–25K and +5.4) in the presence and absence of insulin and Rsg stimulation. Consistent with the previous report that PPARγ is enriched on the –25K but not on the +5.4K enhancer without any stimulation (16), we observed slightly higher binding signals of PPARγ on the enhancer of -25K than on the +5.4K enhancer, and deficiency of Brd4 had no effect on the PPARγ enrichment in the absence of any stimulation (Figure 4E). However, insulin and Rsg stimulated the binding of PPARγ, Brd4, and RNAPII to both enhancers in WT BMDMs (Figure 4E). Insulin and RSG failed to stimulate the recruitment of PPARγ and RNAPII to the enhancers in *Brd4*-deficient BMDMs (Figure 4E), suggesting a Brd4-dependent recruitment of PAPRγ and RNAPII, similar to their recruitments to the promoter of Gdf3 (Figure 4D). These data indicate that PPARγ and Brd4 might have differentially stimulated different enhancers to control the basal and inducible expression of Gdf3 via their bindings to the promoter and enhancers.

Because PPARγ was required for the transcription of *Gdf3*, we also assessed whether PPARγ was required for the recruitment of Brd4 to the promoter and enhancers. As expected, inhibition of PPARγ by its antagonist GW-9662 blocked insulin and Rsg-induced recruitment of PPARγ to the promoter and enhancers of *Gdf3* (Figure 4, F and G). PPARγ inhibition also impaired the recruitment of Brd4 and RNAPII to the promoter and enhancers of *Gdf3* (Figure 4, F and G). Importantly, we found that inhibition of PPARγ by GW-9662 suppressed insulin and Rsg-induced expression of *Gdf3* mRNA and proteins (Figure 4, H and I). Taken together, these data suggest that PPARγ and Brd4 mutually affect signal-dependent recruitment of each other to the promoter and enhancers of *Gdf3* and are essential for the effective RNAPII-dependent transcription of *Gdf3*.

### Brd4-CKO mice have increased expression of lipases and lipolysis in adipocytes due to reduced secreted Gdf3 from ATMs.

**Figure 5:**
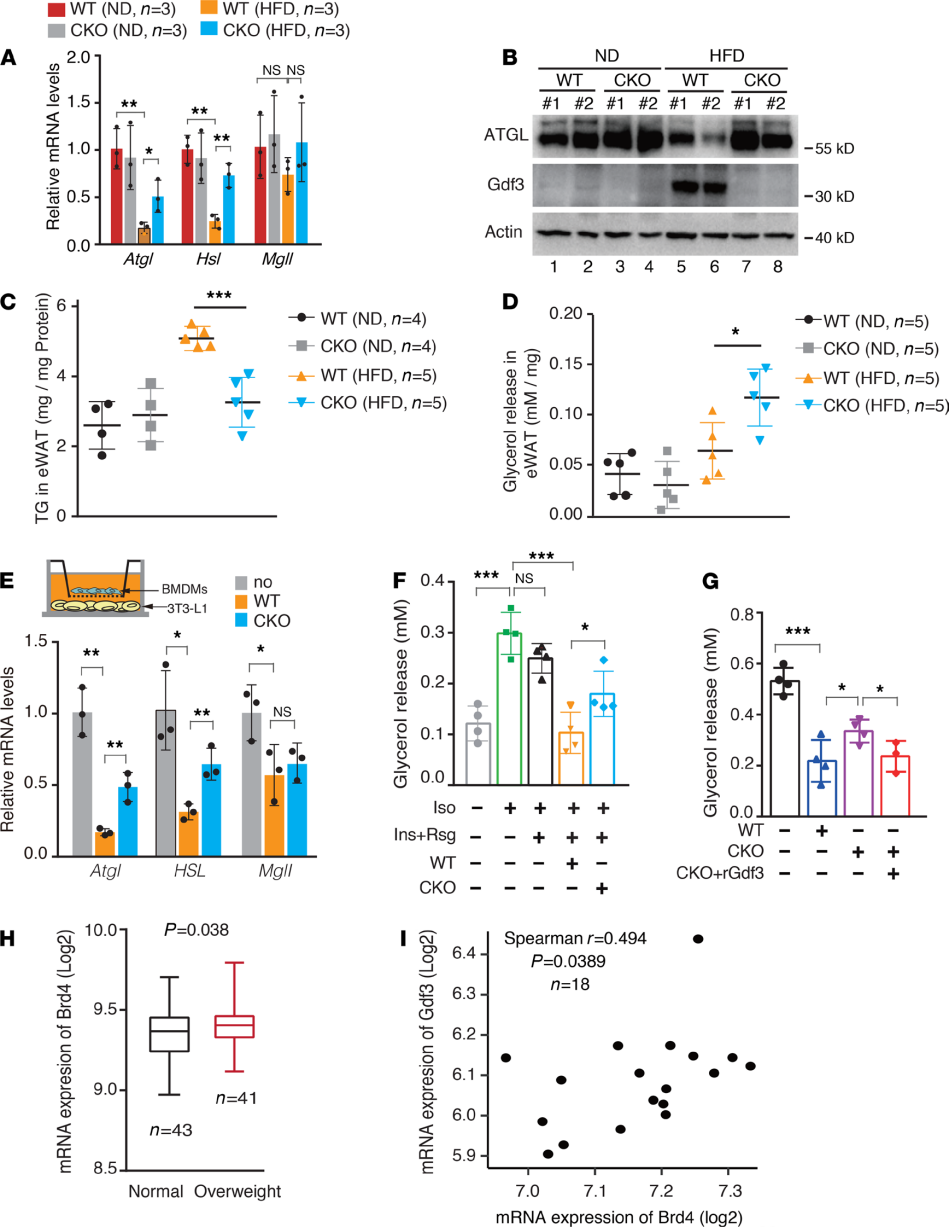
Brd4 increased adiposity through modulating Gdf3-dependent impairment of lipolysis. (**A**) mRNA levels of indicated genes were measured by real-time PCR in eWAT of WT or *Brd4*-CKO mice fed a ND or a HFD for 20 weeks. (**B**) Protein levels of ATGL, Gdf3, and Actin in eWAT of WT or *Brd4*-CKO mice fed a ND or a HFD for 20 weeks were determined by immunoblotting. Each lane represents 1 mouse. ELISA assay of TG (**C**) or glycerol (**D**) levels in eWAT of WT or *Brd4*-CKO mice fed a ND or a HFD for 20 weeks. (**E**) Differentiated 3T3-L1 cells were cocultured without or with WT or *Brd4*-CKO BMDMs as indicated for 12 hours, followed by stimulation with Iso for 3 hours. The mRNA levels of indicated genes in 3T3-L1cells were analyzed by real-time PCR. (**F**) Differentiated 3T3-L1 cells were cocultured without or with WT or *Brd4*-deficient BMDMs with 1 nM insulin and 50 μM Rsg for 12 hours, followed by stimulation with Iso for 3 hours. (**G**) Media collected from WT or *Brd4*-deficient BMDMs treated with insulin and Rsg for 16 hours were incubated with differentiated 3T3-L1 cells for 12 hours, followed by the treatment of Iso for 3 hours. 3T3-L1 cells cultured with media from *Brd4*-CKO BMDMs with the addition of rGdf3 (100 pg/mL) were also shown. (**H**) Analysis of mRNA expression levels of Brd4 in peripheral blood cells from normal weight or overweight individuals from the GEO dataset (GSE109597). (**I**) Correlation of mRNA levels of Brd4 and Gdf3 in circulating monocytes in obese individuals from the GEO dataset (GSE32575). Spearman *r* values and *P* values are indicated. Data are mean and SD and are determined by an unpaired 2-tailed Student’s *t* test (**H**), 1-way (**A**, **C**, **D**, and **E**), or 2-way ANOVA (**F** and **G**). *n =* 2–5 mice as indicated (**A**–**D**). *n =* 3-4 culture dishes from 3–4 independent experiments (**E**–**G**). *P ≤ 0.05, **P ≤ 0.01, ***P ≤ 0.001, ns, statistically not significant. *Brd4*-CKO, myeloid lineage-specific Brd4 knockout; HFD, high-fat diet–induced; ND, normal diet; Iso, isoproterenol; Rsg, rosiglitazone; TG, triglyceride; BMDMs, BM-derived macrophages.

Gdf3 is a known antilipolytic factor released from ATMs and serves as a ligand of ALK7, which activates a signaling cascade to inhibit lipolysis by downregulating the expression of lipases, including ATGL and HSL, the 2 major lipases in eWAT (12–14). We hypothesized that the reduced fat accumulation in the adipose tissue of *Brd4*-CKO mice fed a HFD might result from the increased lipolysis due to the impaired Gdf3 signaling from ATMs. We, therefore, investigated whether Brd4 deficiency in macrophages influenced the expression of these lipases in adipose tissue. Consistent with previous studies (37, 38), mRNA levels of *Atgl* and *Hsl* but not *Mgll* were significantly reduced in eWAT of obese WT mice fed a HFD compared with lean WT mice fed a ND (Figure 5A). However, the mRNA levels of *Atgl* and *Hsl* were partially recovered in eWAT of *Brd4*-CKO mice fed a HFD (Figure 5A). At the protein level, the expression of ATGL was similar in eWAT of WT and *Brd4*-CKO mice fed a ND (Figure 5B). Consistent with the attenuated mRNA expression (Figure 5A), the protein levels of ATGL were decreased in eWAT of HFD-fed WT mice (Figure 5B). Conversely, in HFD-fed *Brd4*-CKO mice, the levels of ATGL protein in eWAT were increased to similar levels of mice fed a ND (Figure 5B). We also measured the Gdf3 proteins in the adipose tissue of these mice and found that the levels of Gdf3 were enriched only in HFD-fed WT obese mice (Figure 5B), indicating an inverse correlation between the expression of Gdf3 and ATGL.

To determine whether the increased ATGL or HSL in eWAT of HFD-fed *Brd4*-CKO mice was associated with enhanced lipolysis, we measured the levels of TGs and released glycerol in eWAT of these mice. On a ND, WT and *Brd4*-CKO mice had similar but relatively low levels of TGs and glycerol (Figure 5, C and D). On a HFD, WT mice had increased TGs in the eWAT (Figure 5C), consistent with the increased fat accumulation during obesity (Figure 1C). Compared with obese WT mice, the levels of TGs were significantly decreased in the eWAT of *Brd4*-CKO mice (Figure 5C). The decreased TGs levels were associated with increased glycerol levels in eWAT of *Brd4*-CKO mice (Figure 5D). The increased glycerol levels likely reflected the reduced inhibition of Gdf3 on the expression of C/EBPα and PPARγ upon excess nutrient because their expression was recovered in eWAT of *Brd4*-CKO mice (Supplemental Figure 10). Collectively, these results suggest that the reduced fat accumulation in *Brd4*-CKO mice resulted from the increased hydrolysis of TGs into glycerol mediated by ATGL and HSL.

To further determine whether Brd4-regulated Gdf3 released from ATMs reduces the expression of ATGL and lipolysis in a paracrine manner, we set up a coculture system in which BMDMs and adipocytes were cocultured in a Transwell plate separated with a filter membrane (Figure 5E). When WT BMDMs were cocultured with mature 3T3-L1 adipocytes in the presence of insulin and Rsg, we observed that isoproterenol-induced (Iso-induced) expression of *Atgl* and *Hsl* but not *Mgll* was reduced in adipocytes (Figure 5E). The expression of *Atgl* and *Hsl* in 3T3-L1 adipocytes was enhanced when cocultured with *Brd4*-deficient BMDMs (Figure 5E). When we measured the released glycerol level from 3T3-L1 adipocytes, we observed that Iso-induced free glycerol levels, which were not affected by insulin and Rsg, were decreased in 3T3-L1 adipocytes when cocultured with WT BMDMs treated with insulin and Rsg (Figure 5F). Compared with coculture with WT BMDMs, the levels of glycerol increased when differentiated 3T3-L1 adipocytes were cocultured with *Brd4*-deficient BMDMs in the presence of insulin and Rsg (Figure 5F), indicating enhanced lipolysis. The partially increased glycerol levels comparing to adipocytes without any cocultured BMDMs likely resulted from the residual Gdf3 activities from *Brd4*-deficient BMDMs (Figure 4, B and C). Furthermore, when we used the media from insulin- and Rsg-treated WT or *Brd4*-CKO BMDMs to stimulate differentiated 3T3-L1 adipocytes, we found that released glycerol levels from adipocytes were significantly higher when cells were stimulated with culture media from *Brd4*-deficient BMDMs (Figure 5G), likely due to the reduced but not completely impaired secretion of Gdf3 from *Brd4*-deficient BMDMs (Figure 5F). Supporting this, addition of recombinant Gdf3 into media from *Brd4*-deficient BMDMs reduced the glycerol levels (Figure 5G). Altogether, these results suggest that Brd4 was essential for the production of Gdf3, which suppresses the expression of lipases and lipolysis in adipocytes.

Finally, we evaluated the relevance of Brd4 and Gdf3 to obesity in human patients. Overweight individuals (BMI ≥ 25) have higher Brd4 expression in blood samples than individuals with normal weight, and the expression levels of Brd4 positively correlate with the expression of Gdf3 in obese individuals (Figure 5, H and I), highlighting the importance of Brd4-Gdf3 regulatory pathway in obesity. However, it has to be noted that a HFD did not alter the protein levels of Brd4 in ATMs (Supplemental Figure 11).

## Discussion

**Figure 6:**
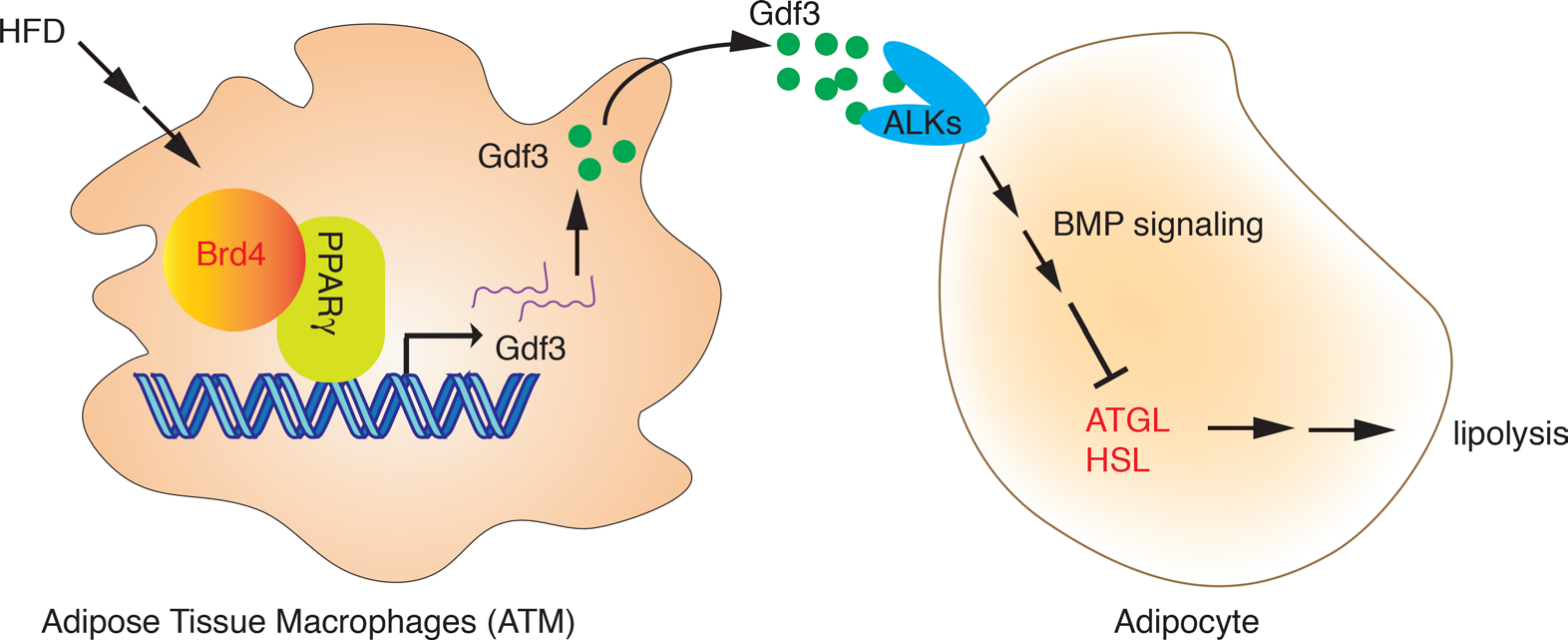
Schematic model for how Brd4 cooperates with PPARγ to activate the expression of Gdf3 in ATMs and suppresses the expression of lipases and the associated lipolysis during diet-induced obesity. ATMs, adipose tissue macrophages.

We have recently shown that Brd4 regulates the innate immune response by facilitating the expression of proinflammatory cytokines in macrophages (28, 39). In this study, we found that Brd4 also regulates the expression of inflammatory cytokines and antilipolytic factors in ATMs, which contribute to fat accumulation, inflammation, and insulin resistance in adipose tissue under excessive nutrients. Specifically, Brd4 cooperates with PPARγ in ATMs to regulate the expression of Gdf3, which acts on the adipocytes to suppress the expression of lipases and lipolysis, resulting in fat accumulation and the development of obesity (Figure 6). Our study establishes Brd4 as an essential metabolic and transcriptional regulator of ATMs and indicates that Brd4 in ATMs could have been a potential therapeutic target for the prevention and treatment of obesity and the associated metabolic diseases.

*Brd4*-CKO mice were resistant to HFD-induced obesity (Figure 1C). The reduced size of adipose tissue was likely attributed to higher metabolic activity with lipid as the major energy substrates in *Brd4*-CKO mice, because *Brd4*-CKO mice fed a HFD had reduced RER and increased energy expenditure (Figure 1, E and F). Accordingly, reduced TG levels and enhanced released glycerol were observed in the eWAT of HFD-fed *Brd4*-CKO mice with increased expression of ATGL and HSL compared with WT mice (Figure 5). ATGL catalyzes the initial step in ATG hydrolysis, working in concert with HSL and other enzymes to mobilize TGs to FFAs for energy production (4). Because mice with overexpression of ATGL in adipocytes have increased fatty acid oxidation (FAO) within adipocytes (8), it is likely that *Brd4*-CKO mice adopted a similar mechanism to increase the use of FFAs for energy production. In line with this, we found that the FAO genes, including *Ech1* and *Cyc1,* were increased in eWAT of HFD-fed *Brd4*-CKO mice compared with HFD-fed WT mice (Supplemental Figure 12A). In the liver and muscle, the expression of *Acc2*, the negative factor of FAO, was decreased, whereas the levels of *Ucp1* and *Ucp2* were enhanced in *Brd4*-CKO mice fed a HFD compared with HFD-fed WT mice (Supplemental Figure 12, B and C). Consistently, the plasma levels of β-hydroxybutyrate, one of the end products of FAO, were increased in HFD-fed *Brd4*-CKO mice (Supplemental Figure 12D), whereas the plasma levels of FFAs and TGs remained relatively unchanged in HFD-fed WT**,** despite increased lipolysis in the adipose tissue (Supplemental Figure 13). These data suggest that increased lipolysis in the adipocytes of *Brd4*-CKO mice was likely associated with increased FAO in liver, muscle, and adipocytes.

Gdf3-ALK7 axis has emerged as a novel interactive mechanism between macrophages and adipocytes in the regulation of adiposity (14, 15, 40). *Gdf3* knockout mice displayed less accumulated adipose tissue than WT mice and showed partial resistance to HFD-induced obesity (40). The level of Gdf3 was significantly decreased in *Brd4*-deficient BMDMs and ATMs (Figure 3, F and G). Similar to *Gdf3* knockout mice, *Brd4*-CKO mice had similar reduced fat accumulation and were resistance to HFD-induced obesity (Figure 1). The Brd4-dependent Gdf3 secretion from macrophages was essential for the inhibition of lipases and lipolysis because adipocytes cocultured with *Brd4*-deficient macrophages had increased expression of ATGL and HSL and elevated hydrolysis of TGs (Figure 5, E and F). Importantly, the culture media from *Brd4*-deficient BMDMs with the addition of recombinant Gdf3 reestablished the inhibitory effect on TG hydrolysis, as evidenced by the reduced release of glycerol (Figure 5G), indicating that the decreased lipolysis in adipocytes is at least partially due to the reduced Gdf3 from *Brd4*-deficient macrophages. Gdf3 then interacts with ALK7 and possibly other receptors, including ALK4 and the activin type receptors, to regulate lipolysis in adipocytes.

Although PPARγ is most abundant in adipocytes and is considered to be the master regulator of adipocyte differentiation and metabolism, PPARγ also regulates the expression of genes in macrophages (41, 42). For example, the expression of Gdf3, which functions as a paracrine signal to control muscle regeneration, is a target gene of PPARγ in macrophages, since the expression of Gdf3 was abolished in PPARγ-deficient BMDMs (16). We found that RNA and protein levels of Gdf3 were diminished in *Brd4*-deficient BMDMs with no change in PPARγ expression compared with WT BMDMs (Figure 4, A and B, and Supplemental Figure 14), raising a possibility that Brd4 might cooperate with PPARγ to stimulate the expression of Gdf3. Indeed, Brd4 and PPARγ occupied the same promoter and enhancer regions of *Gdf3* (Figure 4, D and E). On the one hand, the binding of PPARγ and Brd4 on the promoter of *Gdf3* would facilitate RNAPII binding and the transcription of *Gdf3* (Figure 4). On the other hand, PPARγ and Brd4 might participate in the transcription of Gdf3 by stimulating the synthesis of enhancer RNAs (eRNAs) through their binding to enhancers because both Brd4 and PPARγ could stimulate gene expression via eRNA synthesis in macrophages (22, 43). The binding of Brd4 to the promoter and enhancers appeared to be PPARγ-dependent because inhibition of PPARγ with GW-9662 inhibited the recruitment of Brd4 (Figure 4, F and G). Interestingly, we also found that Brd4 was involved in signal-dependent recruitment of PPARγ, but not the basal level of binding of PPARγ to the promoters and enhancers (Figure 4, D and E). The dependency of Brd4 for the signal-dependent recruitment of PPARγ might result from the intrinsic activities of Brd4 for chromatin decompaction and nucleosome clearance (44). Furthermore, PPARγ is known to regulate the expression of genes involved in lipid metabolism and inflammatory response in macrophages (45–47). Of interest, our RNA-Seq data from ATMs demonstrate that genes involved in inflammatory response or macrophage functions, but not lipid metabolism, are the major group of genes regulated by Brd4 in ATMs (Figure 3). It appears that Brd4 selectively facilitated the expression of a subset of PPARγ target genes in macrophages in a context-dependent manner. For example, the expression of RSG-induced PPARγ genes, including *Angptl4, Ech1, Hadhb*, *Fabp4*, was decreased, whereas the expression of *Cd36, Fabp7*, and *Pnpla2* remained unchanged in *Brd4*-deficient BMDMs (Supplemental Figure 15).

Deficiency of Brd4 in myeloid cells increased insulin sensitivity in mice fed a HFD (Figure 2). Because eWAT and prWAT are linked to insulin resistance and type 2 diabetes (48), the reduced size of WAT from increased lipolysis triggered by Gdf3 reduction might account for the improved insulin sensitivity in *Brd4*-CKO mice (Figure 2). In addition to Gdf3, a significant number of inflammatory genes were altered in *Brd4*-deficient ATMs (Figure 3E). The expression of *Tnfa* and *Il6* were attenuated in ATMs of the HFD-fed *Brd4*-CKO mice (Supplemental Figure 16). Proinflammatory cytokines, including TNF-α and IL-6, are the hallmark of activated ATMs (1, 2). ATMs are activated to release proinflammatory cytokines that amplify the adipose tissue inflammation, resulting in the insulin resistance (1, 2). The impaired ATM activation with less production of TNF-α and IL-6 might have also contributed to the reduced systemic and local inflammation and improved insulin sensitivity in *Brd4*-CKO mice (Figure 2). Besides *Tnfa* and *Il6*, the expression of chemokine receptor Ccr5 was also found to be reduced in the ATMs of *Brd4*-CKO mice (Figure 3E). Ccr5 is critically involved in the infiltration of ATMs in obesity (49). This reduced Ccr5 expression might have resulted in the reduced infiltration of ATMs in *Brd4*-CKO mice because F4/80-positive cells were significantly decreased in the adipose tissue of *Brd4*-CKO mice (Figure 3G and Supplemental Figure 5). Therefore, Brd4 appears to modulate the diverse activities of ATMs, including activation and infiltration, via regulating the expression of various genes. Brd4 might cooperate with distinct transcription factors, such as NF-κB and PPARγ, to differentially regulate the expression of genes in ATMs to modulate the development of obesity and insulin resistance.

In summary, our studies unravel a pathological role of Brd4 in diet-induced obesity and insulin resistance. We also identified a mechanism by which Brd4 stimulates the expression of antilipolytic factor Gdf3 in ATMs via PPARγ. Recent studies reveal that BET inhibitors, including JQ1, exhibit potent antiinflammatory effects in several inflammatory diseases via targeting Brd4-regulated genes in various cells (21–23, 50). We also found that JQ1 suppressed the expression of Gdf3 at both the mRNA level and the protein level in macrophages (Supplemental Figure 17). Therefore, Brd4 could be a target for the treatment of inflammatory metabolic diseases, including obesity. Although various BET inhibitors are undergoing clinical trials for the treatment of cancer and inflammatory diseases (20, 51), it has to be noted that BET inhibitors target all BET family proteins, including Brd2, Brd3, and Brd4. It has been reported that Brd2 disruption in mice causes severe obesity (52). Therefore pan-BET inhibitors might not be suitable for the treatment of obesity and insulin resistance. The Gdf3-ALK7 signaling pathway that is activated in obesity has been indicated as a potential target of medical intervention for obesity (15). It would be of great interest to investigate whether selective inhibition of Brd4 or macrophage Brd4 could affect Gdf3-ALK7 signaling pathway and also be an effective approach to treat obesity and insulin resistance. However, as an important epigenetic transcriptional regulator, Brd4 regulates a mass of gene transcription; besides this, body fat composition and the physiological state of mice are very different from those of humans. Therefore, whether Brd4/Gdf3/Alk7-mediated paracrine signal pathway is feasible and can be applied to clinical practice still needs to be explored and practiced continuously.

## Methods

### Mice and diets.

WT (*Brd4^fl/fl^*) and *Brd4*-CKO (*Brd4^fl/fl^*-*LysM^Cre^*) mice have been previously described (28). Mice were kept under specific pathogen–free conditions at the animal facilities of University of Illinois Urbana-Champaign (UIUC) or Fijian Medical University (FJMU). For all experiments, 4- to 5-week-old male mice were fed a ND (5058, Lab-Diet) or a HFD (nD12451, Research Diets Inc.).

### Antibodies.

Antibodies for immunoblotting were as follows: anti-β-actin (sc-47778, Santa Cruz); anti-β-Tubulin (T8328, Sigma-Aldrich); anti-AKT (9272, Cell Signaling Technology); anti-p-AKT (4051, Cell Signaling Technology); anti-Gdf3 (AF958, R&D Systems); anti-ATGL (sc-8020, Santa Cruz); anti-C/EBPα (sc-365318, Santa Cruz); and anti-PPARγ (sc-7273, Santa Cruz). Antibodies for ChIP were as follows: anti-Brd4 (A301-985A, Bethyl Laboratories Inc); anti-PPARγ (ab41928, Abcam); and anti-RNA polymerase II (ab26721, Abcam). Antibodies for FACS flow cytometry were as follows: anti-mouse CD45.2 PerCP-Cyanine5.5 (45-0454, eBioscience); anti-mouse F4/80 antigen PE (12-4801, eBioscience); anti-mouse CD11b APC-eFluor 780 (47-0112, eBioscience); anti-mouse CD11c Brilliant Violet 60 (117334, BioLegend); and anti-Mouse CD301Alexa Fluor 647 (MCA2392A647, Bio-Rad).

### Measurement of metabolic rate.

Four- to five-week-old male mice fed a HFD for 3 weeks were housed individually in metabolic chambers of the CLAMS (Columbus Instruments). The chamber was maintained at 23°C with 12-hour light/dark cycles, and food and water were available. The first readings were taken after a 48-hour acclimation period. Oxygen consumption rate (VO_2_), carbon dioxide production (VCO_2_) rates, heat production, physical activity, and food and water consumption were measured every 10 minutes over a 48-hour period.

### Measurement of lipid, insulin, and blood glucose.

TG, FFA, glycerol, glucose, and insulin levels were measured with various measuring kits according to the manufacturer’s instructions. Triglyceride Quantification Colorimetric/Fluorometric Kit (K622) was from Biovision; Free Fatty Acid Quantitation Kit (MAK044) was from Sigma; Glycerol Quantitation Kit (MAK117) was from Sigma; and Ultra Sensitive Mouse Insulin ELISA Kit (90080) was from Crystalchem. Blood glucose levels were measured using a blood glucose meter (Accu-Chek Aviva Diabetes Blood Glucose Monitoring System, Roche). For the GTT and ITT, mice were fasted for 6 hours, followed by i.p. injection with glucose (2 g/kg) or human insulin (1 unit/kg).

### Isolation of ATMs from SVCs.

The SVCs of epididymal adipose tissue were prepared as previously described (35). Briefly, epididymal fat pads were weighed, rinsed 3 times in PBS, and then minced in digestion buffer (Hanks’ balanced salt solution with Ca^2+^ and Mg^2+^ supplemented with 0.5% BSA). Tissue suspensions were centrifuged at 500*g* for 5 minutes and then Type II collagenase–treated (1 mg/mL; Sigma-Aldrich, catalog C6885) for 20 minutes at 37°C with shaking. After digestion, EDTA was added to a final concentration of 10 mM and shaken at 37°C for an additional 5 minutes. Cell suspensions were filtered through a 100-μm nylon mesh to separate the ﬂoating adipocyte fraction, and centrifuged for 10 minutes at 500*g*. SVC pellets were then incubated with RBC lysis buffer for 5 minutes at room temperature before centrifugation (10 minutes at 500*g*) and resuspended in FACS buffer (PBS supplemented with 1% FBS and 1 mM EDTA). ATMs from SVCs were purified using a magnetic cell separation system (Miltenyi Biotec) with anti-mouse CD11b microbeads according to the manufacturer’s instructions.

### Flow cytometry analysis.

SVCs were resuspended in FACS buffer and incubated with Fc-Block (BD Bioscience), followed by an incubation with ﬂuorochrome-conjugated primary antibodies. Cells were analyzed using FACS AriaII. Data analysis and compensation were performed using FlowJo. ATMs were gated as CD45^+^F4/80^+^CD11b^+^ cells. Viable CD45^+^F4/80^+^CD11b^+^ ATMs were further identified as M1-ATMs (CD11c^+^CD301^-^) and M2-ATMs (CD11c^-^CD301^+^).

For evaluation of Brd4 expression in ATMs, SVCs stained with ﬂuorochrome-conjugated primary antibodies of CD45, F4/80, and CD11b were then treated with transcription factor staining buffer set (00-5523-00, eBioscience) according to the manufacturer’s instructions. Brd4 was stained using anti-Brd4 (A301-985A, Bethyl Laboratories), followed by incubating with ﬂuorochrome-conjugated secondary antibodies.

### Preparation of BMDMs.

BMDMs were prepared as previously described (28). Briefly, BMs were isolated from the tibia and femur of 8- to 10-week-old male mice. BM cells were cultured in DMEM/F12 supplemented with 10% (vol/vol) heat-inactivated FBS (catalog 10270106, Gibco), 1% Penicillin-Streptomycin (catalog SV30010, Hyclone), L-glutamine (2 mM) (catalog 25030081, Gibco), HEPES buffer (10 mM) (catalog 15630080, Gibco), and 20 ng/mL murine M-CSF (catalog 315-02, PeproTech). The fresh medium was changed on day 4. After 6 days, adherent BMDMs were harvested from plates for experiments.

### Immunoblot of the insulin signaling pathway.

Liver, skeletal muscle, and adipose tissue were collected from WT or *Brd4*-CKO mice sacrificed 5 minutes after intravenous injection with insulin (1 unit/kg). Tissues were immediately homogenized, and cell lysates were immunoblotted for phosphorylated AKT and total AKT with specific antibodies.

### qPCR.

Total RNA from cells or tissues was isolated using the Aurum Total RNA Mini Kit (7326820, Bio-Rad) and was reverse-transcribed using the iScript cDNA Synthesis Kit (170-8891, Bio-Rad). Gene expression was analyzed with iTaq Universal SYBR Green Supermix (172-5124, Bio-Rad). Primer sequences are provided in Supplemental Table 1.

### ELISA.

Levels of cytokines were measured using mouse ELISA Ready-SET-Go! for IL-6 (88-7064, eBioscience), TNF-α (88-7324, eBioscience), and MCP-1 (88-7391, eBioscience). ELISAs were performed according to the manufacturer’s instructions.

### ChIP.

ChIP assay was performed as previously described with minor modifications (28). Briefly, BMDMs were incubated with 1% formaldehyde for 10 minutes at room temperature. The cross-linking was quenched by adding glycine to a final concentration of 125 mM. BMDMs were resuspended in sonication buffers (50 mM Tris-HCl [Tris base, catalog A501492, sangon; HCl, 10011018, sinopharm], pH 8.0, 5 mM EDTA catalog A500895, sangon], and 1% SDS [catalog A100227, sangon]) and sonicated using a Qsonica Q800R2 to reach the desired DNA fragment length (~300–1000 bp). The chromatin sample was precleared by incubation with protein A/G beads for 1 hour and immunoprecipitated with 2 μg of antibody overnight at 4°C. Protein-DNA complex was washed and incubated overnight at 65°C to reverse the cross-linking. DNA was purified for quantitative RT-PCR. The sequences of ChIP primers are provided in Supplemental Table 2.

### RNA-Seq analysis.

Total RNA was extracted from ATMs of WT or *Brd4*-CKO mice fed a HFD for 20 weeks. RNA was checked for quality using Agilent 2100 Bioanalyzer and processed for RNA-Seq by the Beijing Genomic Institute according to their standard protocols. The full dataset is available in the NCBI’s Gene Expression Omnibus (GEO) Datasets (GSE169475).

The raw data were cleaned by removing ligation sequence, low-quality sequence, and repeats with poly-N by homemade scripts. High-quality reads were aligned to the *Mus musculus* reference genome (GCF_000001635.25_GRCm38.p5) using Hierarchical Indexing for Spliced Alignment of Transcripts. The numbers of matched reads were calculated and then normalized by FPKM (expected number of fragments per kilobase of transcript sequence per millions base pairs sequenced). The DEGs were identified between 2 groups if the expression level alteration was above the thresholds of fold change greater than or equal to 2 and adjusted *P* value less than or equal to 0.001. To further understand the biological functions of genes, the identified DEGs were analyzed by gene ontology and KEGG using DAVID. The heat maps of altered genes were generated using the heatmap.2 function of the gplots package.

### Adipocyte differentiation.

3T3-L1 preadipocytes were maintained in DMEM with 10% calf serum and allowed to reach 100% confluence. Cells were differentiated 2 days after confluency (designated as day 0) by the addition of DMEM with 10% FBS supplemented with 5 μg/mL insulin, 1 μM DEX, and 0.5 mM IBMX. After 2 days, cells were grown in DMEM with 10% FBS containing only insulin for another 2 days. Media were changed on day 6 into DMEM with 10% FBS for another 2 days. 3T3-L1 cells were ready for coculture experiments on day 8.

### Coculture of adipocytes with BMDMs.

Cells were cocultured using 12-well plates containing Transwell inserts with a 0.4-μm porous membrane. Differentiated 3T3-L1 adipocytes were seeded at the bottom of the well (5 × 10^5^/well), whereas BMDMs (5 × 10^5^/well) were seeded on the membrane insert. After incubation with DMEM with 10% FBS containing 1 nM insulin and 50 μM rosiglitazone for 12 hours, cells were stimulated with Iso for 3 hours. Supernatants were collected for glycerol measurement. RNA was collected from 3T3-L1 adipocytes for gene expression analysis.

### IHC staining.

For Gdf3 or F4/80 staining, adipose tissue samples were embedded in Tissue-Tek O.C.T. compound (Sakura), frozen in dry ice. Four-μm continuous sections were prepared for immunostaining with Gdf3 antibody (AF958, R&D Systems) or F4/80 antibody (BM4008, Origene). Gdf3 or F4/80 is indicated by brown staining and nuclei are counterstained in blue. For the quantification of immunostaining, 5 different fields were randomly selected, and the areas covered by Gdf3 or F4/80 staining were determined using ImageJ software with IHC analysis toolbox plug-in (http://imagej.nih.gov/ij/plugins/ihc-toolbox/).

### Statistics.

All data were presented as mean ± SD and a *P* value less than or equal to 0.05 was considered statistically significant. Differences in the mean values between 2 groups were assessed by unpaired 2-tailed Student’s *t* test. Differences in mean values in more than 2 groups were determined by 1-way or 2-way ANOVA.

### Study approval.

All the animal experiments were approved by the UIUC or FJMU IACUC.

## Author contributions

X. Hu and LFC designed the experiment. X. Hu, XD, GL, JC, X. He, HS, YHC, and DHK performed the experiments. X. Hu, XD, GL, JC, HS, DHK, X. He, JKK, and LFC analyzed the data. LFC supervised the research. X. Hu and LFC wrote the paper.

## Supplementary Material

Supplemental data

## Version 1 04/08/2021

Electronic publication
