# Supplementary material for: Brd4 modulates diet-induced obesity via PPARγ-dependent Gdf3 expression in adipose tissue macrophages
Source: JCI Insight. 2021 Apr 8;6(7):e143379. doi: 10.1172/jci.insight.143379 (PMC8119191; doi:10.1172/jci.insight.143379)
Supplement: Supplemental data [file jciinsight-6-143379-s152.pdf]

Supplementary Figure legends:

Figure S1. Representative images of adipose tissue of WT or *Brd4*-CKO mice fed ND or HFD for 20 weeks.

Figure S2. Weights of indicated organs from WT or *Brd4*-CKO mice fed ND or HFD for 20 weeks. Data are means and standard deviation and are determined by an unpaired two-tailed Student's t test (left) or one-way ANOVA (right). n=5 mice. ***P≤0.001; ns, statistically not significant.

Figure S3. Metabolic studies of WT or *Brd4*-CKO mice fed HFD measured over 48 hr by CLAMS. (A) Physical activity measurement of ambulatory activity on the X-axis (XAMB), total beam breaks on the X-axis (XTOT), and total beam breaks on the Z-axis (ZTOT). (B&C) Food intake (B) and CO_2 release (C) of WT or *Brd4*-CKO mice fed HFD. Data are means and standard deviation and are determined by an unpaired two-tailed Student's t test. n=4-5 mice. *P≤0.05, **P≤0.01, ***P≤0.001; ns, statistically not significant.

Figure S4. Plasma levels glucose (A) and insulin (B) of fasting WT or *Brd4*-CKO mice upon ND or HFD. Data are means and standard deviation and are determined by one-way ANOVA. n=5 mice. *P≤0.05, ***P≤0.001; ns, statistically not significant.

Figure S5. FACS analysis of stromal vascular cells (SVCs) of eWAT of WT or *Brd4*-CKO mice fed HFD for 20 weeks. (A) The representative plot represents adipose tissue macrophages (ATMs: $CD45^+$ $F4/80^+$ $CD11b^+$). (B) The number of ATMs in eWAT of WT or *Brd4*-CKO mice fed HFD (left). The ratio of ATMs to SVCs (right). Data are means and standard deviation and are determined by an unpaired two-tailed Student's t

test. n=3 mice. **P≤0.01.

Figure S6. Heat map of the relative expression levels of genes from ATMs of HFD-fed WT or *Brd4*-CKO mice (scaled Z-score) based on log2-normalized expression levels of differently expressed genes (DEGs).

Figure S7. WT or *Brd4*-deficient BMDMs were treated with or without 1 nM Ins or 50 μM Rsg for 12 hr, *Gdf3* mRNA levels were analyzed by real-time PCR. Data are means and standard deviation and are determined by one-way ANOVA. n=3 culture dishes from 3 independent experiments. ns, statistically not significant.

Figure S8. Schematic representation of *Gdf3* promoter region with the positions of PPARγ binding site. TSS: transcription start site.

Figure S9. *Cdf3* gene tracks of ChIP-seq peaks for Brd4, PPARγ and histone modification H3K27Ac on chromosome 6, mm10. Y-axis indicated normalized ChIP-seq signals. Seven putative *Gdf3* enhancers are highlighted by boxes. The diagram was obtained by the modification of GEO Datasets (GSE109131 for Brd4, GSE21314 for PPARγ, and GSE106701 for H2K27Ac) of the National Center for Biotechnology Information.

Figure S10. The mRNA and protein levels of C/EBPα and PPARγ were measured by real-time PCR (A) or immunoblotting (B) in eWATs of WT or *Brd4*-CKO mice fed ND or HFD for 20 weeks. Each lane represents one mouse. Data are means and standard deviation and are determined by one-way ANOVA. n=4-5 mice. *P≤0.05, ns, statistically not significant.

Figure S11. Protein levels of Brd4 were detected by flow cytometry in adipose tissue

macrophages (ATMs) of WT mice fed ND or HFD for 20 weeks. Data are means with standard deviation and are determined by an unpaired two-tailed Student's t test. n=3 mice. ns, statistically not significant.

Figure S12. mRNA levels of indicated FAO related genes in eWAT (A), liver (B) and muscle (C) were measured by RT-PCR from WT or *Brd4*-CKO mice fed ND or HFD for 20 weeks. (D) Plasma levels of β-hydroxybutyrate in WT or *Brd4*-CKO mice fed ND or HFD for 20 weeks. All data are means and standard deviation and are determined by one-way ANOVA. n=4 mice. *$P \leq 0.05$, **$P \leq 0.01$, ***$P \leq 0.001$, ns, statistically not significant.

Figure S13. Plasma levels of FFA (A) and TG (B) in the WT or *Brd4*-CKO mice fed ND or HFD for 20 weeks. Data are means and standard deviation and are determined by one-way ANOVA. n=5 mice. *$P \leq 0.05$; ns, statistically not significant.

Figure S14. WT or *Brd4*-deficient BMDMs were treated with or without 0.1, 1, 10 nM Insulin (Ins) and 50 μM rosiglitazone (Rsg) for 16 hr. PPARγ protein levels were analyzed by immunoblotting.

Figure S15. WT or *Brd4* KO BMDMs were treated with or without RSG (50 μM) for 24 h. The mRNA levels of indicated PPARγ-targeted genes were analyzed by RT-PCR. Data are means and standard deviation and are determined by one-way ANOVA. n=3 culture dishes from 3 independent experiments. *$P \leq 0.05$, **$P \leq 0.01$, ns, statistically not significant.

Figure S16. mRNA levels of *Il6* and *Tnfa* in $CD11b^+$ ATMs isolated from WT or *Brd4*-CKO mice fed HFD for 20 weeks. Data are means and standard deviation and are determined by an unpaired two-tailed Student's t test. n=4 mice. ***$P \leq 0.001$.

Figure S17. WT BMDMs were pretreated with Brd4 inhibitor JQ1 (1 μM or 5 μM) for 1 hr, followed by the stimulation with Ins+Rsg for 12 hr. mRNA (A) and protein (B) levels of Gdf3 were analyzed by RT-PCR or immunoblotting, respectively. Data are means and standard deviation and are determined by one-way ANOVA. n=3 culture dishes from 3 independent experiments. **$P \leq 0.01$; ***$P \leq 0.001$; ns, statistically not significant.

S1

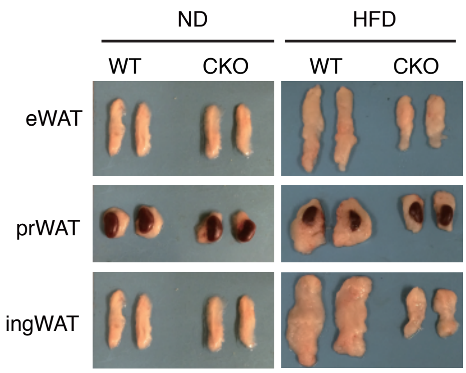
ND
HFD
WT
CKO
WT
CKO
eWAT
prWAT
ingWAT

S2

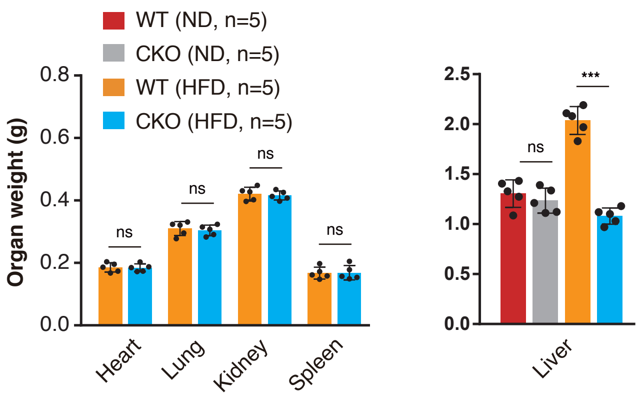
WT (ND, n=5)
CKO (ND, n=5)
WT (HFD, n=5)
CKO (HFD, n=5)
Organ weight (g)
0.8
0.6
0.4
0.2
0.0
ns
ns
ns
ns
Heart
Lung
Kidney
Spleen
2.5
2.0
1.5
1.0
0.5
0.0
ns
***
Liver

S3

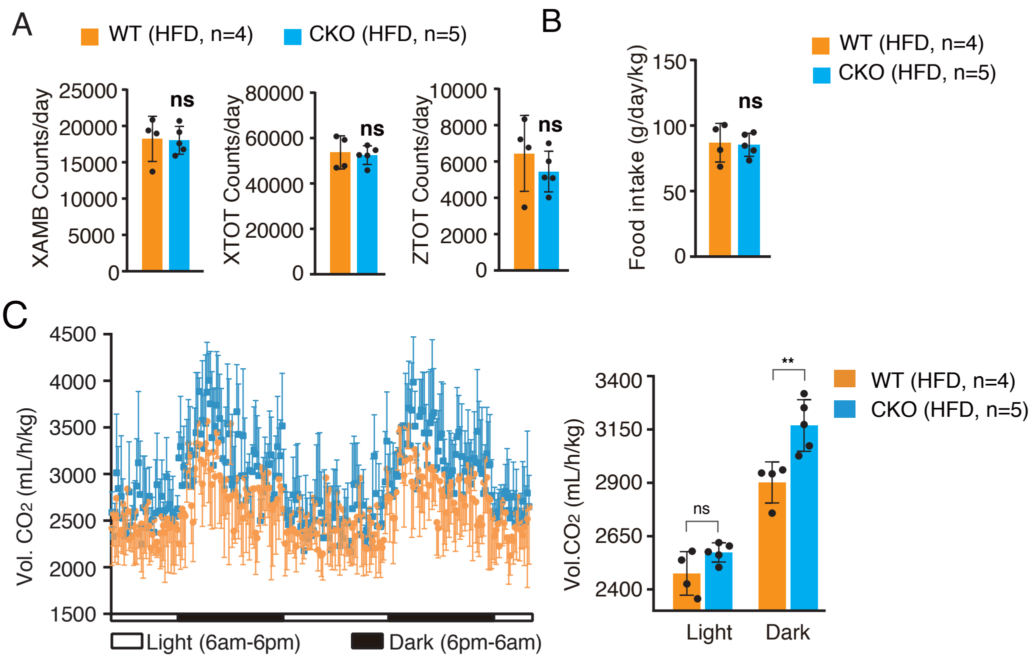
A
WT (HFD, n=4)
CKO (HFD, n=5)
XAMB Counts/day
25000
20000
15000
10000
5000
0
ns
XTOT Counts/day
80000
60000
40000
20000
0
ns
ZTOT Counts/day
10000
8000
6000
4000
2000
0
ns
B
Food intake (g/day/kg)
150
100
50
0
ns
WT (HFD, n=4)
CKO (HFD, n=5)
C
Vol. CO2 (mL/h/kg)
4500
4000
3500
3000
2500
2000
1500
Light (6am-6pm)
Dark (6pm-6am)
Vol.CO2 (mL/h/kg)
3400
3150
2900
2650
2400
ns
**
Light
Dark
WT (HFD, n=4)
CKO (HFD, n=5)

S4

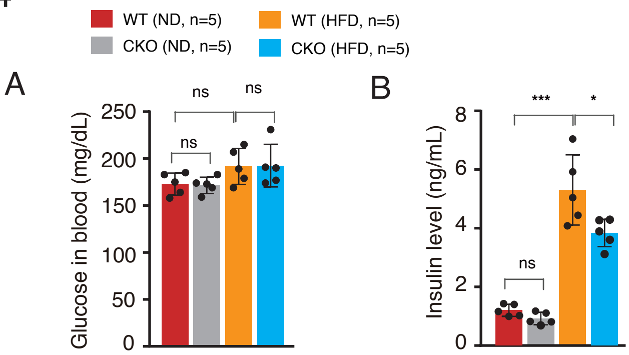
WT (ND, n=5)
CKO (ND, n=5)
WT (HFD, n=5)
CKO (HFD, n=5)
A
Glucose in blood (mg/dL)
250
200
150
100
50
0
ns
ns
ns
B
Insulin level (ng/mL)
8
6
4
2
0
***
*
ns

S5

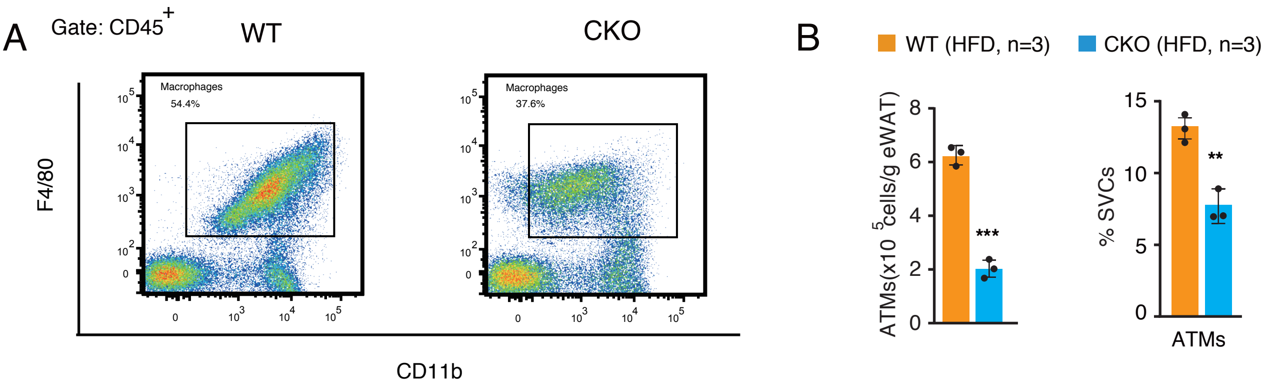
A
Gate: CD45+
WT
CKO
Macrophages
54.4%
Macrophages
37.6%
F4/80
CD11b
B
WT (HFD, n=3)
CKO (HFD, n=3)
ATMs(x10 5cells/g eWAT)
8
6
4
2
0
***
% SVCs
15
10
5
0
**
ATMs

S6

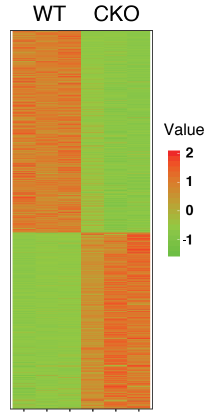

S7

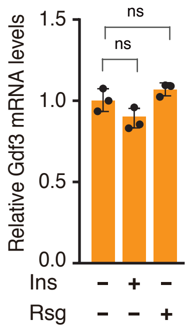

S8

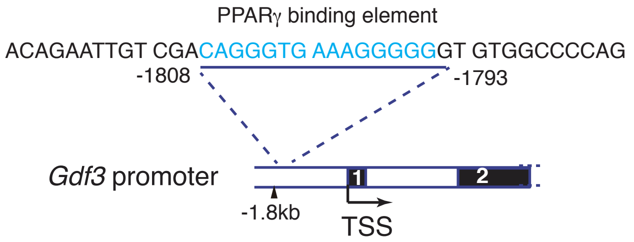

S9

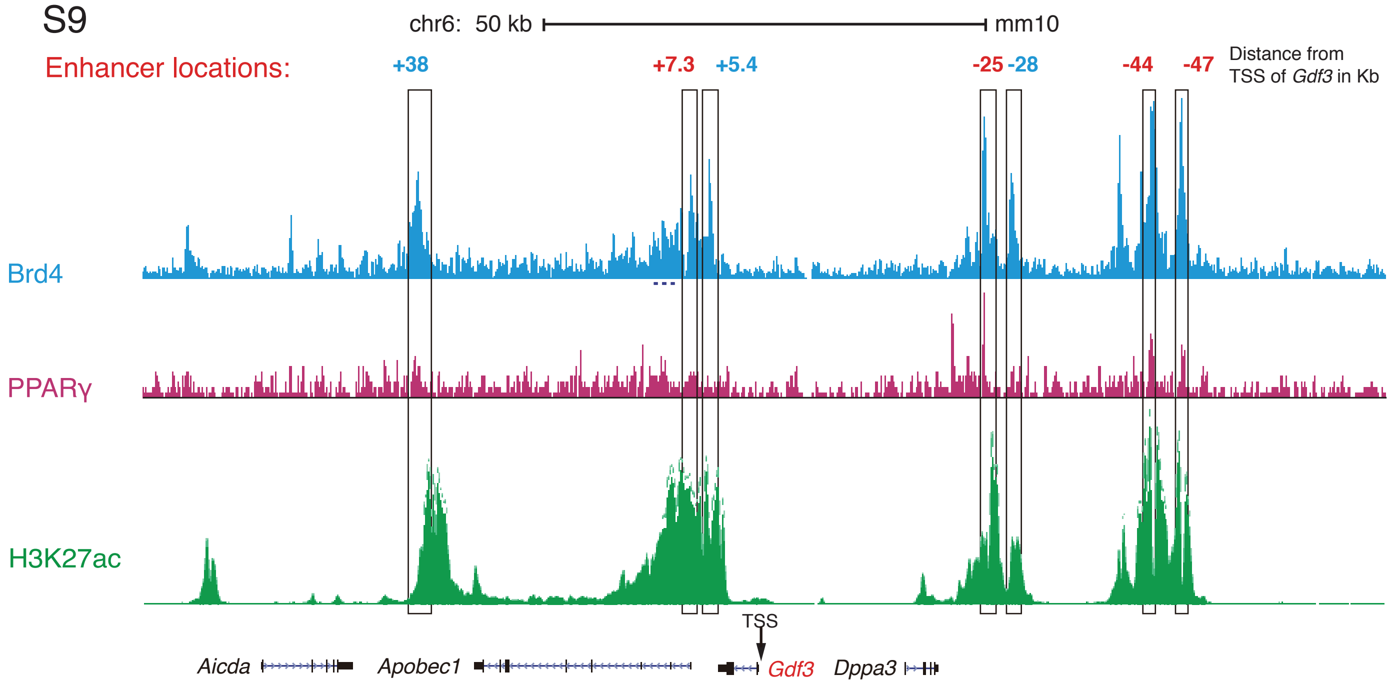

S10

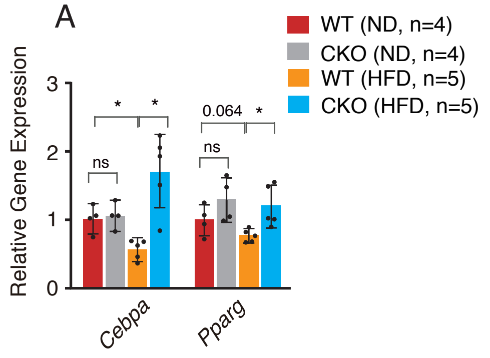

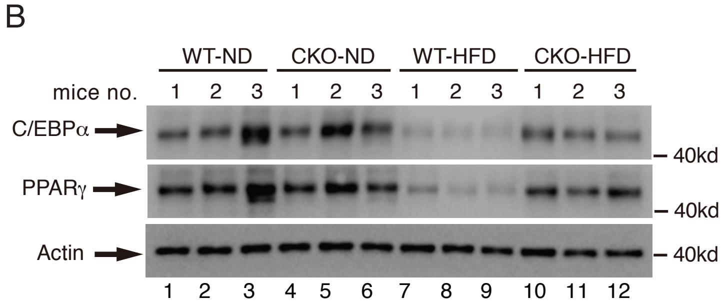

S11

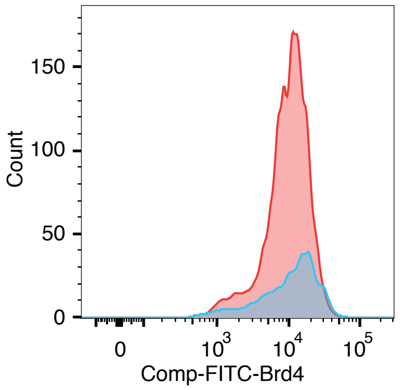

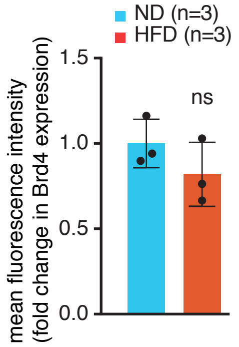

S12

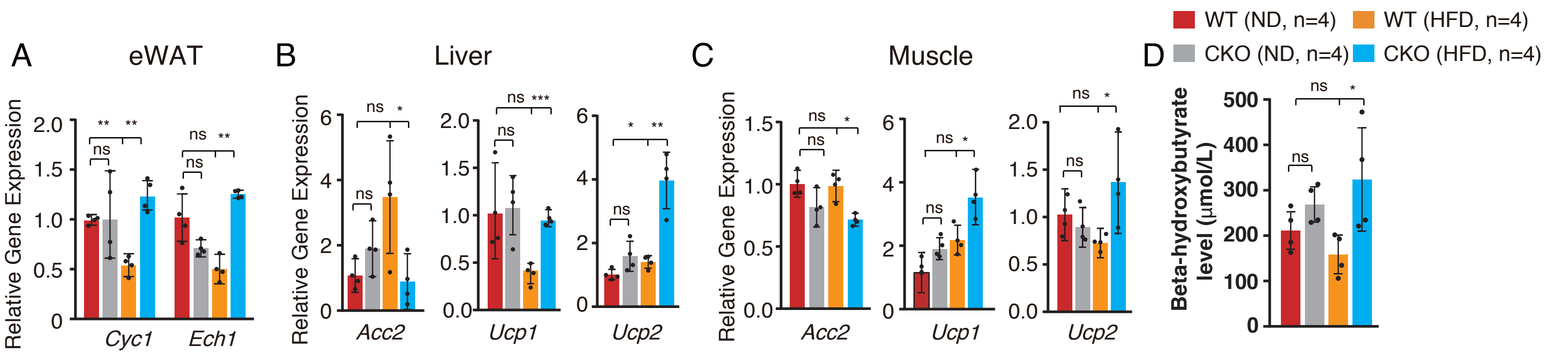

S13

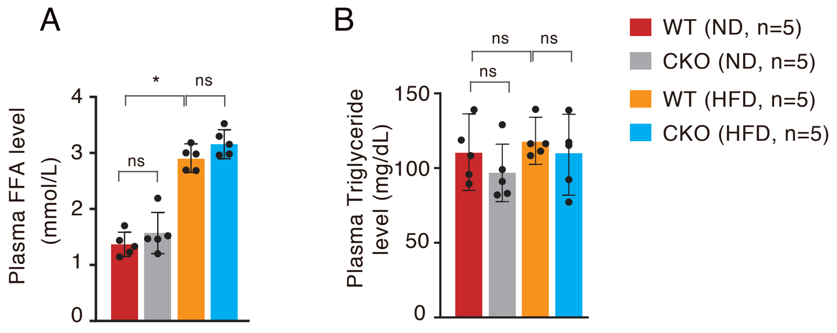

S14

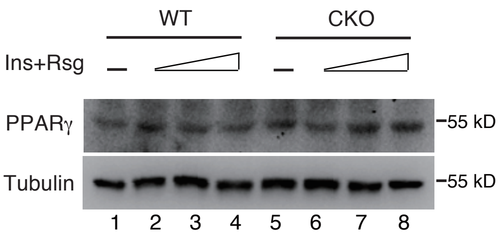

S15

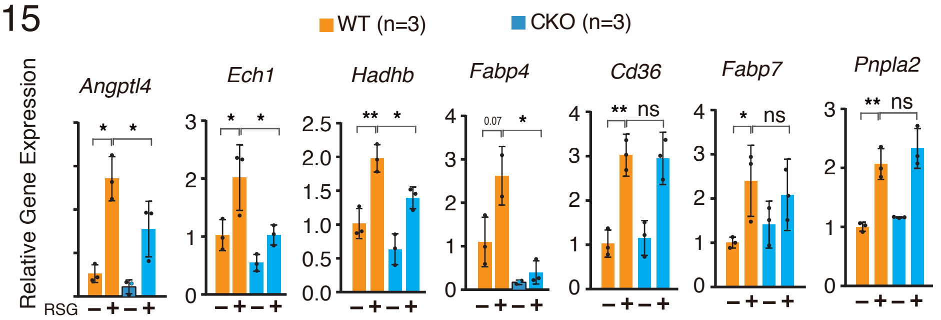

S16

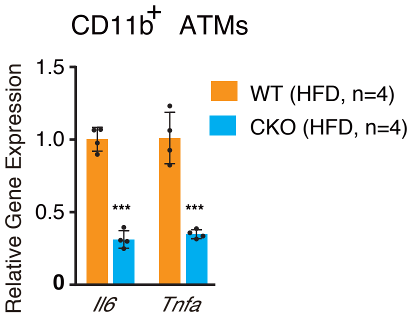

S17

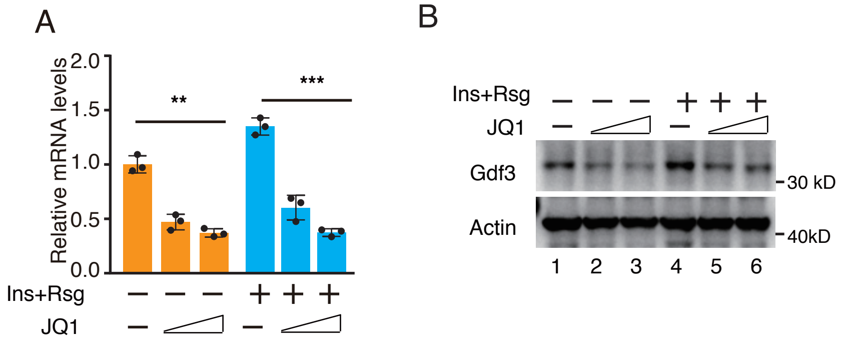

Table 1. Primers used for qRT-PCR

qRT-PCR	Forward (5'-3')	Reverse (5'-3')
Actin	CGGTTCCGATGCCCTGAGGCTCTT	CGTCACACTTCATGATGGAATTGA
Il6	TAGTCCTTCCTACCCCAATTTCC	TTGGTCCTTAGCCACTCCTTC
Tnfa	CCCTCACACTCAGATCATCTTCT	GCTACGACGTGGGCTACAG
Mcp1	CCACTCACCTGCTGCTACTCAT	TGGTGATCCTCTTGTAGCTCTCC
Gdf3	CGAGTTTCAAGACTCTGACC	CACGTAGCATAAGTCCTGCG
Atgl/Pnpla2	TGACCATCTGCCTTCCAGA	TGTAGGTGGCGCAAGACA
Hsl	GCGCTGGAGGAGTGTTTTT	CCGCTCTCCAGTTGAACC
Mgll	CGGACTTCCAAGTTTTTGTCAGA	GCAGCCACTAGGATGGAGATG
Cebpa	CAAGAACAGCAACGAGTACCG	GTCACTGGTCAACTCCAGCAC
Pparg	CCAGAGTCTGCTGATCTGCG	GCCACCTCTTTGCTCTGATC
Cyc1	CAGCTTCCATTGCGGACAC	GGCACTCACGGCAGAATGAA
Ech1	GCTACCGCGATGACAGTTTC	TCAGAGATCGAAGGCTGATGTT
Acc2	CGCTCACCAACAGTAAGGTGG	GCTTGGCAGGGAGTTCCTC
Ucp1	AGGCTTCCAGTACCATTAGGT	CTGAGTGAGGCAAAGCTGATTT
Ucp2	ATGGTTGGTTTCAAGGCCACA	CGGTATCCAGAGGGAAAGTGAT
Angptl4	CATCCTGGGACGAGATGAACT	TGACAAGCGTTACCACAGGC
Hadhb	ACTACATCAAAATGGGCTCTCAG	AGCAGAAATGGAATGCGGACC
Fabp4	AAGGTGAAGAGCATCATAACCCT	TCACGCCTTTCATAACACATTCC
CD36	ATGGGCTGTGATCGGAACTG	GTCTTCCCAATAAGCATGTCTCC
Fabp7	GGACACAATGCACATTCAAGAAC	CCGAACCACAGACTTACAGTTT

Table 2. Primers used for ChIP-qPCR

ChIP	Forward (5'-3')	Reverse (5'-3')
Gdf3 (Promoter)	TCTCAACTAAAGCTAGAACC	GACAATTCTGTGCTTTTCCAC
Gdf3 (-25k Enhancer)	GCTCTGTAGATCTCCCAAGGTTG	CTGTGGTGAGGCAGCAAGTCATG
Gdf3 (+5.4k Enhancer)	GTGCACGCCTTTAATCCCAGCAC	GTGTTCTGCAAACACCTCTCAC
